# *Arabidopsis* HOT3/eIF5B1 constrains rRNA RNAi by facilitating 18S rRNA maturation

**DOI:** 10.1073/pnas.2301081120

**Published:** 2023-04-03

**Authors:** Runlai Hang, Ye Xu, Xufeng Wang, Hao Hu, Nora Flynn, Chenjiang You, Xuemei Chen

**Affiliations:** ^a^Department of Botany and Plant Sciences, Institute for Integrative Genome Biology, University of California, Riverside, CA 92521; ^b^College of Life Sciences, South China Agricultural University, Guangdong Laboratory for Lingnan Modern Agriculture, Guangzhou, Guangdong 510642, China; ^c^School of Life Sciences, Peking-Tsinghua Joint Center for Life Sciences, Peking University, Beijing 100871, China

**Keywords:** HOT3, eIF5B, rRNA, risiRNA, RDR1

## Abstract

Ribosome biogenesis is essential for protein synthesis in gene expression. Yeast eIF5B has been shown biochemically to facilitate 18S ribosomal RNA (rRNA) 3′ end maturation during late-stage 40S ribosomal subunit assembly and gate the transition from translation initiation to elongation. But the genome-wide effects of eIF5B have not been studied at the single-nucleotide resolution in any organism, and 18S rRNA 3′ end maturation is poorly understood in plants. *Arabidopsis* HOT3/eIF5B1 was found to promote development and heat stress acclimation by translational regulation, but its molecular function remained unknown. Here, we show that HOT3 is a late-stage ribosome biogenesis factor that facilitates 18S rRNA 3′ end processing and is a translation initiation factor that globally impacts the transition from initiation to elongation. By developing and implementing 18S-ENDseq, we revealed previously unknown events in 18S rRNA 3′ end maturation or metabolism. We quantitatively defined processing hotspots and identified adenylation as the prevalent nontemplated RNA addition at the 3′ ends of pre-18S rRNAs. Aberrant 18S rRNA maturation in *hot3* further activated RNA interference to generate RDR1- and DCL2/4-dependent risiRNAs mainly from a 3′ portion of 18S rRNA. We further showed that risiRNAs in *hot3* were predominantly localized in ribosome-free fractions and were not responsible for the 18S rRNA maturation or translation initiation defects in *hot3*. Our study uncovered the molecular function of HOT3/eIF5B1 in 18S rRNA maturation at the late 40S assembly stage and revealed the regulatory crosstalk among ribosome biogenesis, messenger RNA (mRNA) translation initiation, and siRNA biogenesis in plants.

## Significance

Translation initiation is tightly coupled with late-stage ribosome biogenesis in yeast and mammals by eIF5B. However, the global effects of eIF5B at single-nucleotide resolution have not been studied in any organism, and little is known about 18S ribosomal RNA (rRNA) maturation in plants. Here, by developing the quantitative 18S-ENDseq method, we defined the role of *Arabidopsis* HOT3/eIF5B1 in 18S rRNA 3′ end maturation. We uncovered the genome-wide role of HOT3 in gating the transition from translation initiation to elongation. Aberrant 18S rRNA maturation in *hot3* was accompanied by the production of rRNA-derived siRNAs in an RDR1- and DCL2/DCL4-depedent manner. Our findings highlight the regulatory crosstalk among mRNA translation initiation, ribosome biogenesis, and siRNA biogenesis in plants.

Protein synthesis by messenger RNA (mRNA) translation is a key step in gene expression that involves a large number of ribosomes (1, 2). The eukaryotic 80S ribosome comprises a 40S small subunit (SSU) and a 60S large subunit (LSU). The 40S SSU contains one copy of 18S ribosomal RNA (rRNA) and over 33 ribosomal proteins, while the 60S LSU consists of one copy each of 25S/28S, 5.8S, and 5S rRNAs, as well as over 46 ribosomal proteins (1). Ribosome biogenesis is a complex progress coordinated by cellular RNA polymerases including Pol I, Pol II, and Pol III (3, 4). Ribosomal DNA (rDNA) transcription by Pol I in the nucleolus and by Pol III in the nucleoplasm generates primary transcripts, which undergo diverse covalent RNA modifications and dynamic ribosome assembly coupled with pre-rRNA processing with the help of snoRNAs and over 200 ribosome biogenesis factors. Transcribed spacers such as the 5′ external transcribed spacer (ETS), internal transcribed spacer 1 (ITS1), ITS2, and 3′ ETS are removed before the final maturation of 40S and 60S ribosomal subunits in the cytoplasm (*SI Appendix*, Fig. S1*A*) by multiple processing events representing dynamic ribosome assembly described in different species (5–9).

As an energy-consuming process, ribosome biogenesis is tightly surveilled and precisely regulated (10). Defects in pre-rRNA processing, like other aberrant RNA processing/decay events, may trigger RNA interference (RNAi), effectively causing a self-reinforcing cycle of RNA degradation and small interfering RNA (siRNA) production. In *Caenorhabditis elegans*, aberrant rRNA biogenesis is associated with the production of rRNA-derived siRNAs (risiRNAs), which are loaded into Argonaute (AGO) proteins to inhibit Pol I transcription and silence pre-rRNAs via nucleolar RNAi (11–13), thereby maintaining rRNA homeostasis. risiRNA biogenesis is suppressed by a functional PIWI-interacting RNA pathway (14) and by polyuridylation and degradation of rRNA (15) in *C. elegans* and by Trf–Air–Mtr4 polyadenylation complex–mediated nuclear RNA surveillance in *Schizosaccharomyces pombe* (16). In *Arabidopsis thaliana*, aberrant rRNA processing events in *fiery1* (*fry1*) and *xrn2 xrn3* mutants are accompanied by risiRNA biogenesis in an RDR6 (RNA-dependent RNA polymerase 6)- and DCL2 (Dicer-Like2)/DCL4-dependent manner, and the risiRNAs compete with microRNAs (miRNAs) for AGO1 occupancy (17).

Ribosome biogenesis at the late pre-40S assembly stage in the cytoplasm is tightly coordinated with translation initiation to prevent premature interactions between preribosomes and other translational apparatus components to ensure translational accuracy (18). The final cleavage at the D site in pre-18S rRNA by NOB1 [NIN1 (RPN12) binding protein 1 homolog] to release the mature 18S rRNA converts pre-40S particles into functional 40S subunits (*SI Appendix*, Fig. S1*A*) (19, 20), and this step is promoted by translation initiation factor eIF5B/Fun2-dependent quality control in yeast (21–24). In *Arabidopsis* late-stage 40S assembly, the 18S-A2 precursor generated by endonucleolytic cleavage at the A2 site in ITS1 or processed from the 18S-A3 precursor (*SI Appendix*, Fig. S1*A*) is proposed to be trimmed by the exonuclease RRP6L2 (25) before 18S rRNA maturation, probably by AtNOB1-mediated cleavage at the D site in the cytoplasm (8, 26, 27). The 60S-associated ribosome biogenesis factor large 60S subunit nuclear export GTPase 1-2 (LSG1-2) promotes late 40S maturation (28). Consequently, 18S-A2 intermediates (also known as 20S rRNA) accumulate in an *AtNOB1* cosuppression line (27), as well as in loss-of-function mutants such as *lsg1-2*, *lsg1-1−/+ lsg1-2−/−* (28), and *rrpl6l2* (25).

In canonical cap-dependent mRNA translation initiation, the 5′ 7-methylguanosine 5′-triphosphate cap of an mRNA is first recognized by the eIF4F complex, which further recruits other eukaryotic initiation factors (eIFs) to form the preinitiation complex (PIC), which scans the mRNA 5′ untranslated region in the 5′ to 3′ direction to search for the AUG initiation codon (29, 30). Later, eIF5B promotes the transition from translation initiation to elongation (31, 32). It first binds to the PIC and recruits the 60S LSU along with the release of eIFs. Finally, eIF5B-GTP hydrolysis triggers the release of eIF5B-GDP and eIF1A, yielding the functional 80S ribosome competent for translation elongation (21, 33, 34). eIF5B and its prokaryotic counterpart IF2 are one of the two universally conserved initiation factors across Bacteria, Archaea, and Eukarya (21). Research in the past half a century highlighted the importance of eIF5B as a multifunctional initiation factor to coordinate 40S SSU maturation, Met-tRNAi stabilization, 60S LSU recruitment, and ribosome-dependent GTPase activity required for the formation of translation-competent 80S (21). In *Saccharomyces cerevisiae*, knocking out eIF5B led to severely slowed growth (35). Dysfunction of eIF5B in *Drosophila melanogaster* results in lethality at the larval stage (36). In *Arabidopsis thaliana*, HOT3/eIF5B1 is required for both development and heat stress acclimation by translational regulation, as uncovered by polysome profiling and RNA-seq analysis (37). Interestingly, *hot3-2* mutants exhibited a slightly lower ratio of 18S to 25S rRNAs (37). However, much remains enigmatic about the molecular function of HOT3 in ribosome biogenesis and mRNA translation in plants.

Here, we uncovered *Arabidopsis* HOT3 as a late-stage ribosome biogenesis factor that coordinates 18S rRNA 3′ end processing and mRNA translation initiation at the genome-wide scale. Loss of function of HOT3 reduced the abundance of mature 18S rRNA and caused genome-wide ribosomal stalling at the ATG translation start codon. By developing and using 18S-ENDseq, we quantitatively defined pre-18S rRNA processing hotspots in ITS1 and found adenylation as the prevalent nontemplated RNA tailing event at the 3′ ends of pre-18S rRNAs. Aberrant 18S rRNA maturation in *hot3* further activated RNAi to generate risiRNAs mainly from a 3′ portion of 18S rRNA. The biogenesis of risiRNAs in *hot3* requires the RNAi machinery, including RDR1, DCL2, and DCL4. We further showed that risiRNAs in *hot3* were predominantly localized in ribosome-free fractions and were not responsible for the 18S rRNA maturation or translation initiation defects of *hot3*. Collectively, our findings uncovered the molecular function of HOT3 in 18S rRNA maturation at the late 40S assembly stage and revealed the regulatory cross talk among ribosome biogenesis, mRNA translation initiation, and siRNA biogenesis in plants.

## Results

### *HOT3* Is Required for Ribosome Biogenesis in *Arabidopsis*.

**Fig. 1.**
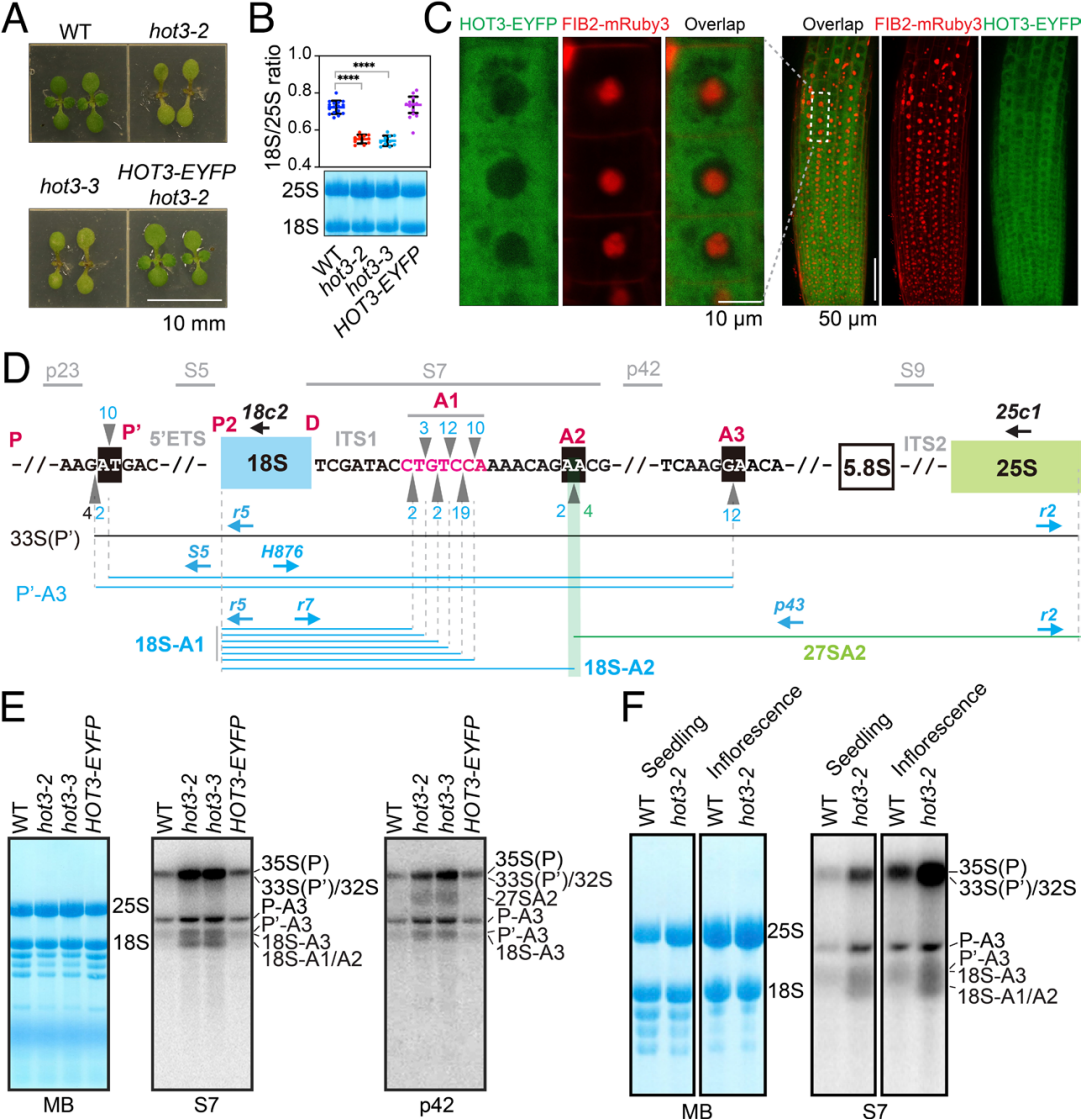
Cytoplasmic HOT3 is required for proper pre-rRNA processing. (*A*) *Top* views of 10-d-old seedlings on MS medium (Scale bar, 10 mm). *HOT3-EYFP* is driven by the *HOT3* promoter. (*B*) Quantification of the ratio of 18S:25S rRNAs. A representative membrane stained with methylene blue (MB) for total seedling RNAs from WT, *hot3-2, hot3-3*, and *HOT3*::*HOT3-EYFP hot3-2* (*HOT3-EYFP*) is shown. More than 10 independent RNA samples on membranes were stained with MB for quantification. Mean with SD is shown. The unpaired parametric *t* test was used for the evaluation of significant differences between each genotype and WT. ****P < 0.0001. (*C*) Imaging of HOT3-EYFP in root cells by confocal microscopy. FIB2-mRuby3 is a nucleolus marker. Roots of 7-d-old T1 transgenic seedlings of *FIB2::FIB2-mRuby3* in the *HOT3::HOT3-EYFP* *hot3-2* background were imaged. The pictures in the *Left* panel were captured at a higher magnification by rescanning the region marked by the dotted rectangle in the *Right* panel. (*D*) Schematic representation of rDNA and pre-rRNA processing intermediates. The mature 18S and 25S regions are represented by the blue and green rectangles, respectively. 5′ ETS, 5′ external transcribed spacer; ITS1, internal transcribed spacer 1; ITS2, internal transcribed spacer 2. The black line represents 33S(P′), while the blue and green lines represent various processing intermediates. Probe oligonucleotides used for northern blotting are shown at the top in gray. Key processing sites in the 5′ ETS and ITS1 are shown above the DNA sequence in red. Circular RT-PCR primers are represented by blue arrows. The primers are as follows: r5/r2 for 33S(P′), S5/H876 for P′-A3, r5/r7 for 18S-A1/A2, and p43/r2 for 27SA2. The reverse transcription primers for circular RT-PCR are 18c2 and 25c1: 18c2_cDNAs served as the templates for PCR with primer pairs S5/H876 and r5/r7; 25c1_cDNAs were used as templates for PCR with primer pairs r5/r2 and p43/r2. The gray triangles represent the ends of pre-rRNAs as defined by circular RT-PCR clones, and the numbers of clones are shown above the gray triangles. Pre-18S rRNAs with 3′ ends mapped to 8 to 13 nucleotides to the right of the D site are collectively referred to as 18S-A1. (*E* and *F*) Pre-rRNA northern blots with probes in the ITS1 region. The blots of S7 and p42 share the same membrane in (*E*). MB, methylene blue staining. The bands representing pre-rRNA processing intermediates are indicated to the right of the blots and diagrammed in (*D*) and *SI Appendix*, Fig. S1*A*.

To explore the molecular function of *HOT3* in ribosome biogenesis in *Arabidopsis*, we obtained and characterized two T-DNA insertion mutants, *hot3-2* and *hot3-3* (37). RNA sequencing confirmed the sites of T-DNA insertion (37), as evidenced by the lack of reads near the insertion sites (*SI Appendix*, Fig. S2*A*). Both mutants exhibited similar developmental defects (Fig. 1*A*) (37), indicating that both mutations compromised *HOT3* function. RNA sequencing identified a higher number of up-regulated genes than down-regulated genes in the two *hot3* mutants (*SI Appendix*, Fig. S2*B*). Gene ontology analysis for the 1,292 up-regulated genes shared by both alleles revealed translation and ribosome biogenesis as the top two enriched terms (*SI Appendix*, Fig. S2 *C* and *D*). Upregulation of translation- or ribosome-related genes was frequently reported in ribosome biogenesis–related mutants (38, 39). This result indicates that ribosome biogenesis may be affected in *hot3* mutants. Consistent with an earlier report (37), an apparent decrease in the ratio of 18S:25S rRNAs in *hot3* alleles was observed by both methylene blue (MB) staining and bioanalyzer analysis with total RNAs (Fig. 1*B* and *SI Appendix*, Fig. S3). Abnormal morphological phenotypes and the reduced 18S:25S rRNA ratio in *hot3-2* were rescued by genetic complementation with *HOT3*::*HOT3-EYFP* (Fig. 1 *A* and *B* and *SI Appendix*, Fig. S3), suggesting that *HOT3* contributes to 18S rRNA biogenesis. Consistent with the canonical function of eIF5B in cytoplasmic translation (21), HOT3-EYFP was mainly localized in the cytoplasm (Fig. 1*C*), where the final 18S rRNA maturation occurs (26).

To elucidate which step of 18S rRNA biogenesis was impaired in *hot3*, northern blotting was performed with WT, *hot3* alleles, and *HOT3::HOT3-EYFP hot3-2* (referred to hereafter as *HOT3-EYFP*) to detect pre-rRNA processing intermediates. Probes in 5′ ETS and ITS1 regions flanking the mature 18S rRNA (Fig. 1*D* and *SI Appendix*, Fig. S1*A* and Table S1), as described before (39, 40), were used. Aberrant 18S rRNA maturation was detected, as evidenced by overaccumulation of pre-18S rRNAs in *hot3*, including P-A3 detected by p23 (*SI Appendix*, Fig. S4*A*), P′-A3 detected by S5 (*SI Appendix*, Fig. S4*A*), and 18S-A2 detected by S7 (Fig. 1*E*). Using a circular RT-PCR assay (38, 41, 42), we validated most of the accumulated pre-rRNAs mentioned above in *hot3-2* (*SI Appendix*, Fig. S4 *B* and *C*) and further identified higher levels of 33S(P′) (*SI Appendix*, Fig. S4*C*), which was not well distinguished from other early pre-rRNA transcripts by northern blotting (Fig. 1*E* and *SI Appendix*, Fig. S4*A*). Sequencing of the circular RT-PCR products revealed the 5′ and 3′ ends of the various pre-rRNA processing intermediates (Fig. 1*D*). The A2 site is an endonucleolytic cleavage site characterized in WT (43), and 18S-A2 (also known as 20S) was always thought to be the processing intermediate detected by probes between sites D and A2 in WT (8, 44). Of note, circular RT-PCR showed that the major population of pre-18S processing intermediates below the 18S-A3 band detected by probe S7 in *hot3* mutants (Fig. 1*E*) consisted of a series of processing intermediates ending 8-13 nt to the right of the D site (Fig. 1*D*). In fact, pre-18S processing intermediates with heterogeneous 3′ boundaries between sites D and A2 have been reported in *Arabidopsis* and rice wild-type backgrounds (9, 25). Hereafter, we refer to the processing hotspots between D and A2 collectively as A1 (Fig. 1*D*) and pre-18S rRNAs with 3′ ends at A1 and A2 as 18S-A1 and 18S-A2, respectively, or 18S-A1/A2 collectively (Fig. 1 *D* and *E*). The number of clones for 18S-A1 is much more than that for 18S-A2 (Fig. 1*D*), indicating 18S-A1 rather than 18S-A2 may be the major pre-18S intermediate detected by the probe S7. Northern blotting also detected higher levels of 27S rRNAs by probes p42 and S9 (Fig. 1 *D* and *E* and *SI Appendix*, Fig. S4*A*) and pre-5.8S rRNAs by probe S9 (*SI Appendix*, Fig. S4*A*). It indicates that HOT3 may facilitate the maturation of 18S, 5.8S, and 25S rRNAs directly or that HOT3 acts primarily on 18S rRNA maturation, but the status of cytoplasmic processing of 18S-A1/A2 is communicated to the nucleus to affect the maturation of 5.8S and 25S rRNAs indirectly. We prefer the latter possibility as HOT3 is localized to the cytoplasm. In addition, although 25S and 18S rRNAs share the primary transcript, effects of HOT3 on rRNA maturation is stronger for 18S than 25S rRNAs, which may account for the decreased 18S:25S ratio in *hot3*. Aberrant pre-rRNA processing and overaccumulation of 18S-A1/A2 precursors also existed in inflorescences of *hot3-2* (Fig. 1*F*). Therefore, *HOT3* is required for 18S rRNA maturation, especially at the transition from 18S-A1/A2 to 18S rRNA.

**Fig. 2.**
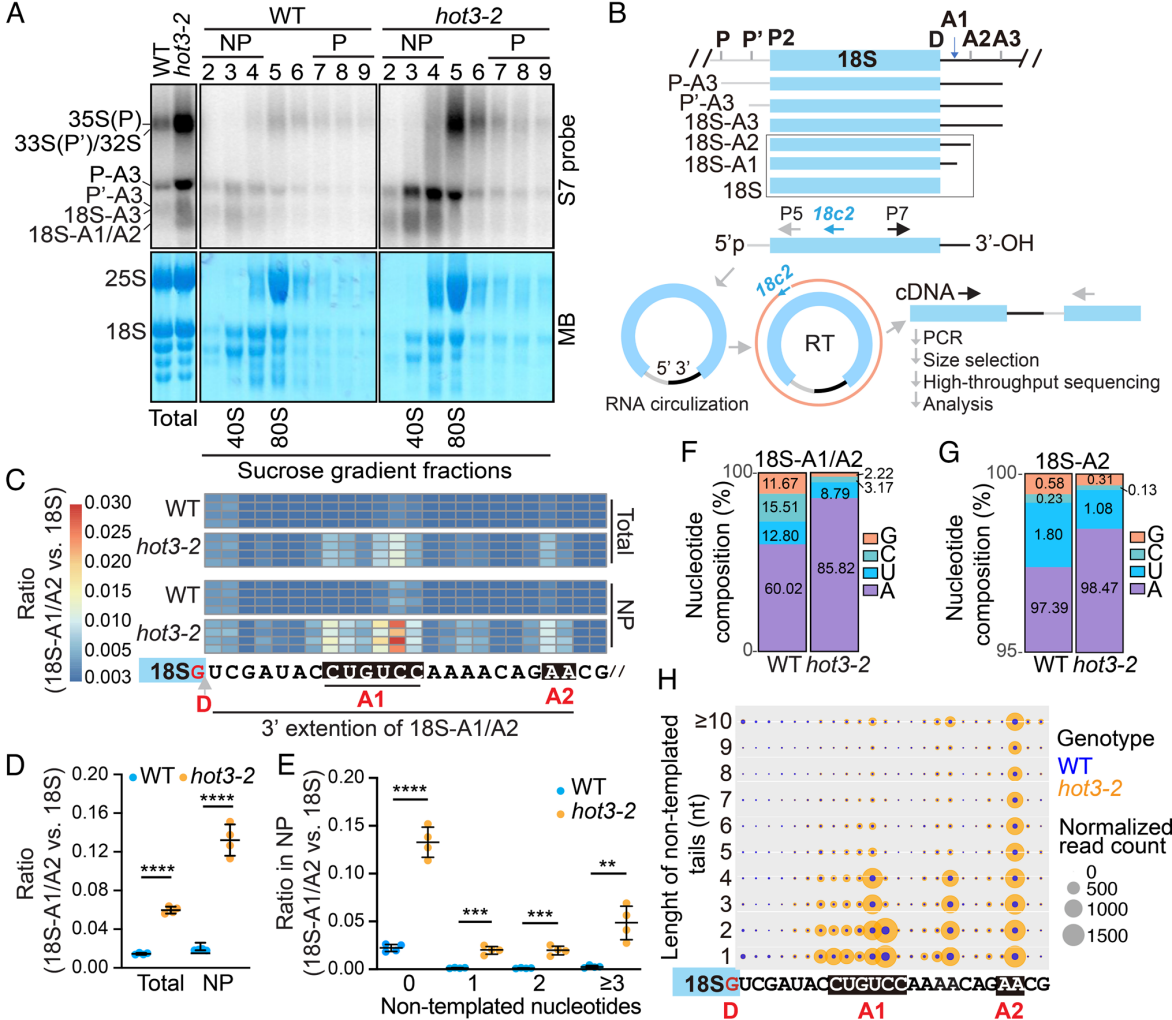
HOT3 promotes 18S rRNA 3′ end maturation. (*A*) Distribution of pre-18S intermediates detected by probe S7 in sucrose gradient fractions of WT and *hot3-2*. The MB staining indicates the patterns of 25S rRNA in the 60S ribosome LSU and 18S rRNA in the 40S ribosome SSU. NP, 80S ribosome-free fractions. P, polyribosome fractions. The samples before fractionation (Total) are shown on the *Left*. (*B*) Pipeline of 18S-ENDseq library construction. Circularized pre-18S rRNAs by ligation with T4 RNA ligase 1 are reverse-transcribed with 18c2 into first-strand cDNA. The P5 universal primer (P5) and P7 indexed primers (P7) are used to amplify 5′ and 3′ flanking sequences in the head-to-tail manner. Barcoded libraries containing chimeric extremities are pooled and sequenced to determine and quantify the 18S ends in different genotypes. (*C*) Heat map to show the 3′ ends of 18S and pre-18S rRNAs as determined by 18S-ENDseq. Reads with 3′ ends mapped to the D site and in the ITS1 region are categorized as 18S and pre-18S, respectively. The ratio of pre-18S:18S rRNAs is shown. Each row represents an independent replicate. Processing sites D, A1, and A2 are indicated. The arrow indicates the position of the 3′ end of mature 18S rRNA. Total, cell lysate before sucrose gradient fractionation; NP, nonpolysome fractions. (*D* and *E*) Quantification of the ratio of pre-18S:18S rRNAs in total and nonpolysome (NP) fractions (*D*) and quantification of the ratio of pre-18S species with nontemplated nucleotides versus mature 18S species in NP fractions (*E*). The unpaired nonparametric *t* test was used to evaluate significance of differences. The *P* values are indicated as follows: 0.001 ≤ *P* < 0.01 (**), 0.0001 ≤ *P* < 0.001 (***), and *P* <0.0001 (****). (*F*) Nucleotide composition of nontemplated tails of pre-18S and 18S rRNA species in WT and *hot3-2* nonpolysome fractions. (*G*) Nucleotide composition of nontemplated tails of 18S-A2 rRNA species in WT and *hot3-2* nonpolysome fractions. (*H*) Bubble plot to show length and normalized read counts of nontemplated nucleotides tailing pre-18S and 18S rRNA species in WT (blue) and *hot3-2* (orange). The *x* axis indicates the 3′ end of the perfectly matched portion of each read, and the *y* axis indicates the length of nontemplated tails. The size of each bubble shows the normalized read counts of the corresponding RNA species.

To determine whether the accumulated pre-18S rRNA processing intermediates in *hot3* were incorporated into translating polysomes, we applied sucrose gradient ultracentrifugation to fractionate endogenous ribosomes from total lysates of *Arabidopsis* seedlings. Fraction 5 in WT and *hot3-2* represented 80S monosomes as it exhibited highly abundant 25S rRNA in the 60S LSU and 18S rRNA in the 40S SSU (Fig. 2 *A*, *Bottom*). Fraction 5 might also contain preribosome particles (such as the 90S processome) that are intermediates in ribosome biogenesis in the nucleolus (45–47), as evidenced by the presence of early pre-rRNA transcripts 35S(P), 33S(P′), and 32S in both wild type and *hot3* (Fig. 2 *A*, *Top*). Fractions 2 to 4 and 7 to 9 were taken to approximate nonpolysome (NP) and polysome (P) fractions, respectively (Fig. 2*A*). Like 20S (18S-A2) rRNA enriched in 40S SSU fractions in yeast (23), the 18S-A1/A2 species were much more abundant in NP than P fractions in both wild type and *hot3-2* (Fig. 2 *A*, *Top*), suggesting that pre-40S SSUs with incomplete pre-18S rRNA processing were not incorporated into translating ribosomes.

### HOT3 Promotes 18S rRNA 3′ End Maturation.

To better characterize and quantify pre-18S rRNA intermediates during 3′ end maturation in wild type and to document the defects in *hot3*, we developed **18S** rRNA **END seq**uencing (18S-ENDseq) by combining circular RT-PCR and high-throughput sequencing (Fig. 2*B*). In brief, circularized pre-18S intermediates were reverse-transcribed using primer 18c2, followed by PCR amplification using customized primers containing the Illumina P5 and barcoded P7 sequences. PCR was performed with limited cycles to prevent saturation. Barcoded PCR products were size-selected and pooled for high-throughput sequencing. RNAs from total lysates (Total) and nonpolysome (NP) fractions from wild-type and *hot3-2* seedlings were subjected to 18S-ENDseq. Only pre-18S rRNA intermediates exhibiting a 5′ end at the P2 site (Fig. 2*B*), which is the 5′ end of the mature 18S rRNA, were further analyzed, thereby excluding reads from other 5′ ETS-containing pre-18S rRNA species. For quantitative comparison among different libraries, we normalized reads of 18S-A1/A2 species against mature 18S rRNA with the 3′ end mapped to the D site (Fig. 2*C*).

Four replicates of 18S-ENDseq libraries gave consistent results showing that incompletely processed 18S rRNA with 3′ ends beyond the D site were more abundant in *hot3-2* than in wild type (Fig. 2 *C* and *D*, Total). This finding was congruous with the trends observed in circular RT-PCR experiments (Fig. 1*D* and *SI Appendix*, Fig. S4*B*), but 18S-ENDseq was more quantitative due to the higher sequencing depth. These incompletely processed 18S rRNAs in *hot3-2* had 3′ ends in two clusters in the ITS1 region between D and A2 sites, with A1 and A2 being the major and minor clusters, respectively (Fig. 2*C*, Total). Thus, incompletely processed 18S-A1/A2 species accumulated in *hot3-2* as compared to wild type. The accumulation of 18S-A1/A2 in *hot3-2* was more severe in the nonpolysome fraction (Fig. 2 *C* and *D*), consistent with findings from northern blotting (Fig. 2*A*). Notably, 18S-A1 was not previously annotated as a processing intermediate, although they were detected by 3′ rapid amplification of cDNA ends (3′ RACE) (25). Having found a higher abundance of 18S-A1 than 18S-A2 in *hot3-2*, we wondered whether this was also the case in wild type. 18S-ENDseq results showed that 18S-A1 species were also present at a higher level than 18S-A2 in wild type (*SI Appendix*, Fig. S5), suggesting that they are intermediates or by-products in pre-18S processing.

Nontemplated adenylation and uridylation of pre-rRNA processing intermediates were reported to be functional in 18S rRNA maturation or by-product elimination in *Arabidopsis* and *C. elegans* (25, 48). Our 18S-ENDseq also revealed nontemplated nucleotide tails at the 3′ ends of 18S-A1/A2. To explore whether these nontemplated nucleotide additions were affected in *hot3,* we evaluated the tailing status for 18S-A1/A2 using datasets from NP as the NP fraction showed higher levels of 18S-A1/A2 (Fig. 2*C*). In wild type, little nontemplated nucleotide addition was found for 18S-A1/A2, but levels of 18S-A1/A2 species with one, two, or more nontemplated nucleotides added to the 3′ ends were significantly increased in *hot3-2* (Fig. 2*E*). Adenosine was the predominant nontemplated nucleotide for 18S-A1/A2 in wild type and more so in *hot3-2* (Fig. 2*F*). For 18S-A2, adenosine accounted for nearly all nucleotides in the nontemplated tails in both wild type and *hot3-2* (Fig. 2*G*). The nontemplated tails of 18S-A2 appeared longer than those of 18S-A1 (Fig. 2*H*). Thus, 18S-ENDseq uncovered aberrant 18S rRNA 3′ end processing in *hot3-2* and revealed adenylation as the major RNA nontemplated tailing event of processing intermediates. Based on knowledge of plant RNA exosome complexes that act in 18S rRNA 3′ end maturation (48), we speculate that adenylation of 18S-A2 may help the 3′ to 5′ processing of 18S-A2 to 18S-A1. The final step in 18S rRNA 3′ end maturation is the cleavage of the 3′ short tail of 18S-A1 (perhaps also of 18S-A2) by AtNOB1 (27), and the overaccumulation of 18S-A1/A2 in *hot3-2* suggests that this step is facilitated by HOT3. The adenylation of 18S-A1/A2 may also promote the degradation of these aberrant rRNA species.

### Dysfunction of *HOT3* Triggers RNAi to Produce risiRNAs Mainly from the 18S rRNA 3′ Region.

**Fig. 3.**
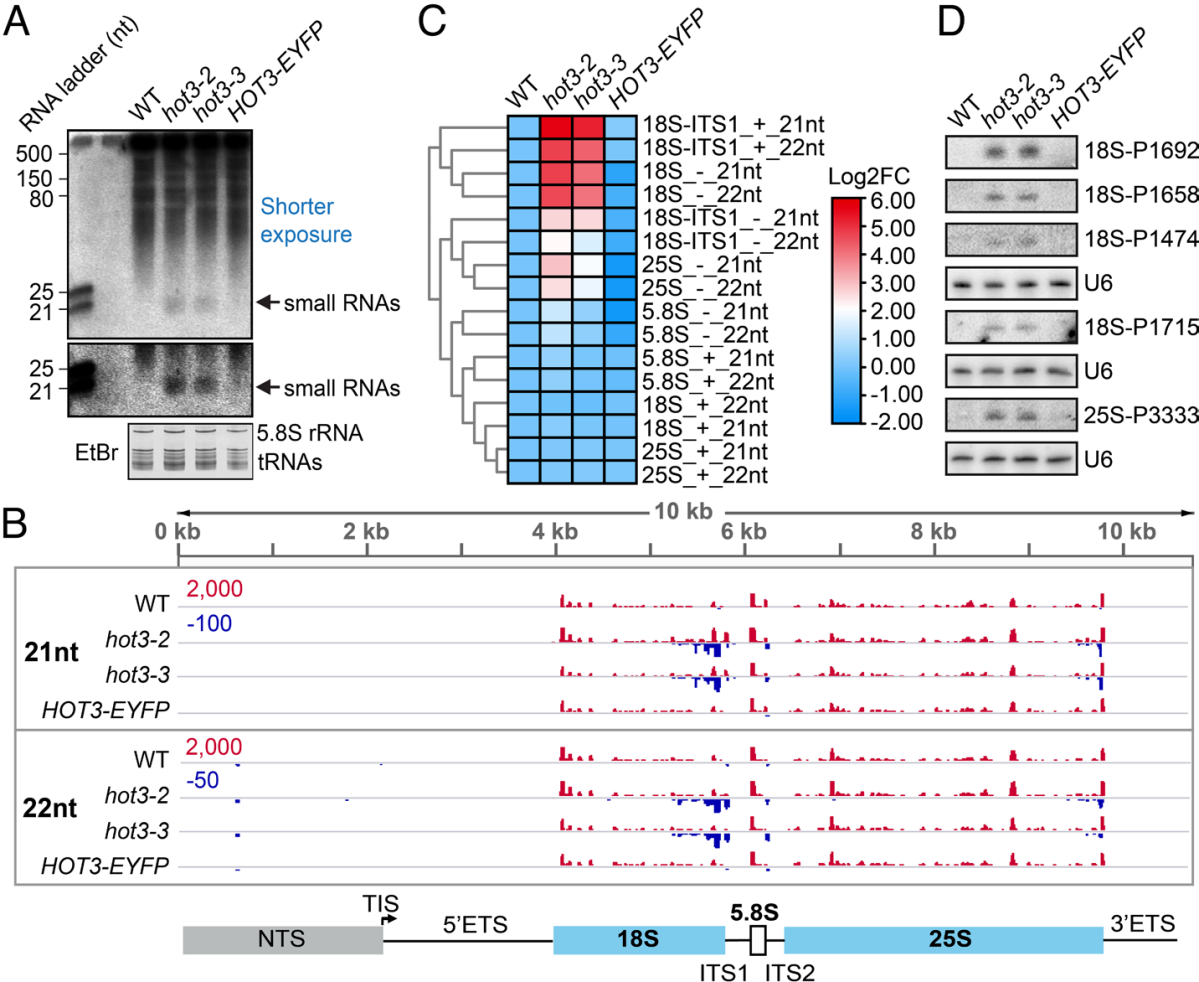
Dysfunction of HOT3 triggers risiRNA biogenesis mainly from the 18S rRNA 3′ region. (*A*) Northern blotting to detect small RNAs from 18S rRNA. Total RNA was resolved in a 15% urea polyacrylamide gel. A randomly labeled 600-bp probe corresponding to the 3′ region of 18S rRNA (amplified by R069/R070 primers in *SI Appendix*, Table S1 and diagrammed in *SI Appendix*, Fig. S1*B*) was used. The 5.8S rRNA and tRNAs in the gel stained with ethidium bromide (EtBr) are shown to indicate relative loading. *HOT3-EYFP,* the transgenic line *HOT3::HOT3-EYFP hot3-2.* (*B*) A genome browser view of 21-nt and 22-nt small RNAs mapped to the rDNA region in WT, *hot3-2, hot3-3*, and *HOT3::HOT3-EYFP hot3-2* (*HOT3-EYFP*). The red and blue peaks represent small RNAs mapped to the positive and the negative strands, respectively. Note that the maximal values on the *y* axis are indicated by the numbers in red and blue for the positive and negative strands, respectively. “TIS” in the rDNA diagram represents the transcription initiation site. NTS, nontranscribed spacer. (*C*) Heat map showing the relative abundance of small RNAs at different positions of the rDNA region. RPM values of small RNAs in *hot3-2, hot3-3*, and *HOT3-EYFP* were compared to those of WT to generate the fold change. The “+” and “−” represent the positive and the negative strands, respectively. “18S-ITS1” represents the region covering both 18S and ITS1. (*D*) Northern blot validation of risiRNAs from 18S rRNA (P1474, P1658, P1692, and P1715) and 25S rRNA (P3333). Blots of P1474, P1658, and P1692 share the same membrane. U6 was used as an internal loading control. P1474, P1658, P1692, P1715, and P3333 are DNA oligonucleotide probes (see *SI Appendix*, Table S1 for their sequences and *SI Appendix*, Fig. S1*B* for their locations in rDNA).

To detect potential products from the degradation of 18S-A1/A2, we used a 600-bp probe corresponding to a 3′ portion of the mature 18S rRNA (*SI Appendix*, Fig. S1*B*) to observe low–molecular weight RNAs. Surprisingly, we observed clear signals of approximately 21 nucleotides (nt) in *hot3* alleles, and the signals disappeared in the *HOT3-EYFP* complementation line (Fig. 3*A*). The small RNAs (sRNAs) could be RNA degradation products from the 18S (or 18S-A1/A2) sense strand, or they may be Dicer products, which would correspond to both strands. To examine this further, we performed sRNA sequencing using total RNAs from WT, *hot3-2*, *hot3-3*, and the *HOT3-EYFP* complementation line in *hot3-2*, with 2 replicates for each genotype. As shown in *SI Appendix*, Fig. S6, the replicates were reproducible, and the sRNAs were concentrated in the 21-nt to 24-nt size range that is characteristic of sRNAs in *Arabidopsis* (17), suggesting that the experiments were successful (*SI Appendix*, Fig. S6). As there are more than five hundred rDNA copies in the *Arabidopsis* haploid genome (4, 49), routine analysis usually generates a high frequency of multiply mapped reads that cannot be assigned to a specific locus. For our purposes, however, there was no need to examine specific rDNA loci. Therefore, for mapping of sRNA reads, we used a 45S rDNA reference sequence widely adopted in *Arabidopsis* research (50–54), which comprises the nontranscribed spacer (NTS), 5′ ETS, 18S, ITS1, 5.8S, ITS2, 25S, and 3′ ETS regions (Fig. 3*B*). All reads that mapped to the rDNA reference were used for subsequent analysis.

We found that much more 21-nt and 22-nt sRNAs were mapped to the antisense strand of rDNA in *hot3* alleles than in wild type (Fig. 3*B*). The sRNAs were mainly derived from a 3′ portion of 18S rRNA, although some sRNAs were also present at the 3′ end of 25S rRNA (Fig. 3*B*). It indicates that aberrant accumulation of rRNA precursors of 18S and 25S rRNAs in *hot3* may trigger sRNA biogenesis. We further quantified the sRNAs based on the subregions and the two strands of 45S rDNA. It was clear that 21-nt and 22-nt sRNAs from the antisense strand of 18S and 25S rRNAs were at higher levels in the two *hot3* alleles and that 21-nt and 22-nt sRNAs from both strands of the 18S-ITS1 linker region were also more abundant (Fig. 3*C*). To validate the presence of sRNAs in *hot3* mutants, we designed DNA oligonucleotide probes to target specific sRNAs derived from the antisense strand of 18S or 25S rRNA (*SI Appendix*, Fig. S1*B*). Northern blotting using these probes validated the accumulation of sRNAs in the two *hot3* alleles (Fig. 3*D*).

The 21-nt and 22-nt size of the sRNAs and the presence of sRNAs derived from the antisense strand of rRNA suggested that the sRNAs in *hot3* mutants were the products of DCL proteins. In fact, the sRNAs were reminiscent of risiRNAs found in *fry1* mutants, which also exhibit aberrant pre-rRNA processing (17). In general, siRNA biogenesis from a single-stranded RNA precursor entails the synthesis of double-strand RNAs by an RDR protein with the help of SUPPRESSOR OF GENE SILENCING3 (SGS3) (55–57) and the digestion of the double-stranded RNAs by DCL2 and DCL4 to generate 22-nt and 21-nt siRNAs, respectively (58, 59). For example, RDR6, SGS3, and DCL4 are required for the production of risiRNAs in *fry1* mutants (17) as well as siRNAs from the *TAS3* locus (60). The biogenesis of *TAS3*-derived siRNAs also involves miR390 (60–62). RDR1 is required for the biogenesis of virus-activated siRNAs (vasiRNAs) including siRNAs from host mRNAs and rRNAs to confer host resistance against viral infection (63). Coding gene–derived siRNAs in *fry1* are also RDR1 and DCL2/4 dependent (17).

**Fig. 4.**
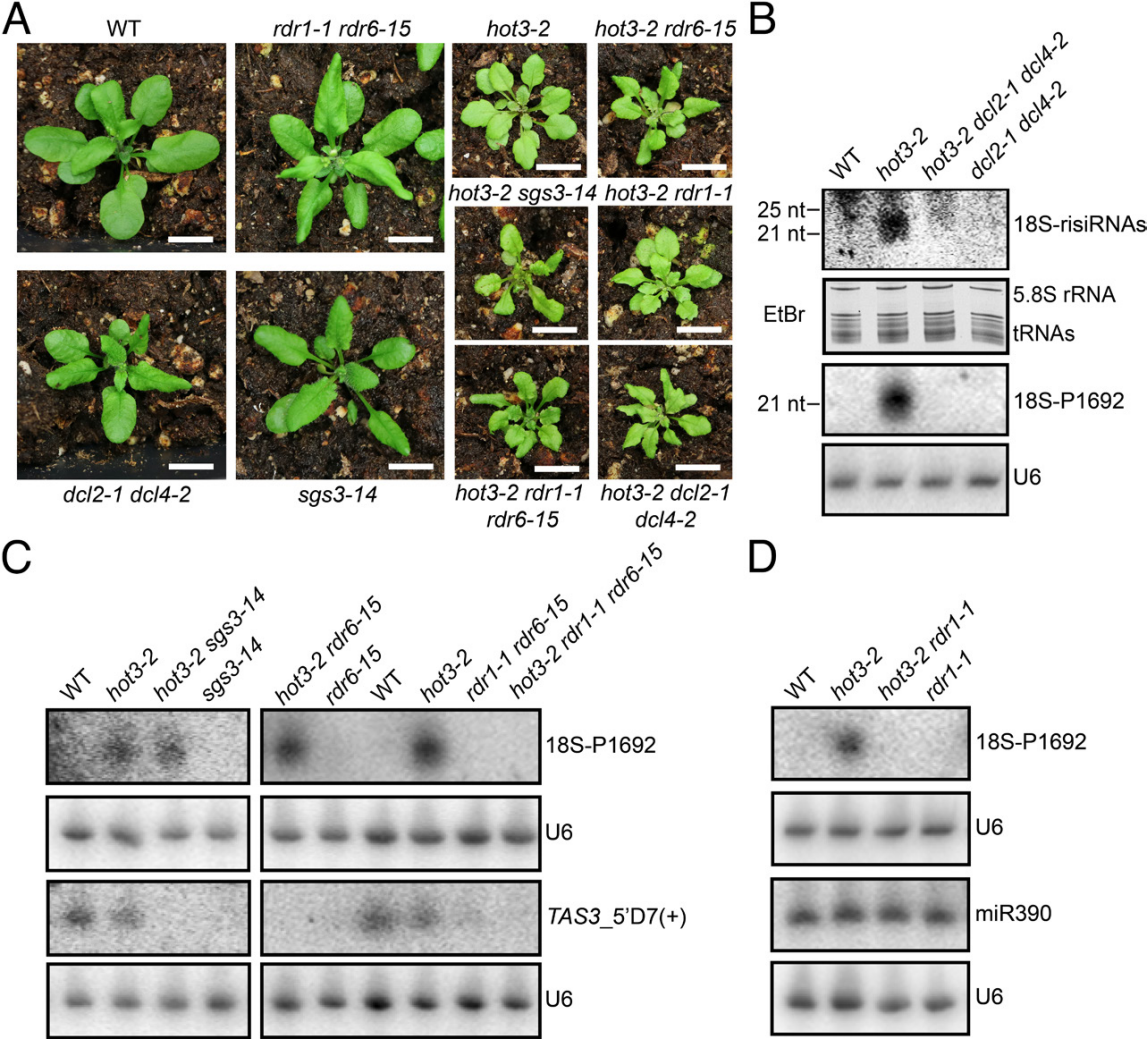
RDR1 and DCL2/4 are required for risiRNA biogenesis in *hot3*. (*A*) 30-d-old seedlings of the indicated genotypes grown in soil. 15-d-old seedlings were transferred from MS medium into soil, and photographs were taken 15 d later (Scale bars, 10 mm). (*B*–*D*) Northern blotting to detect risiRNAs from 18S rRNA in various genotypes. “18S-risiRNAs” (*B*) refers to risiRNAs detected by a 600-bp probe corresponding to the 3′ region of 18S rRNA (amplified by R069/R070 primers in *SI Appendix*, Table S1 and diagrammed in *SI Appendix*, Fig. S1*B*), while “18S-P1692” (*C* and *D*) refers to a risiRNA detected by the P1692 oligonucleotide probe (see *SI Appendix*, Table S1 for its sequence and *SI Appendix*, Fig. S1*B* for its location in rDNA). The RNA gel in (*B*) stained by ethidium bromide (EtBr) before RNA membrane transfer was used to indicate relative loading for the 18S-risiRNAs blot. Each U6 blot serves as a loading control for the corresponding blot above. In (*C*), TAS3-5′D7(+), which is dependent on SGS3 and RDR6 for biogenesis, was included as a control. In (*D*), miR390, whose biogenesis does not require RDR1, was included for comparison.

To clarify whether the sRNAs in *hot3* mutants were indeed risiRNAs, we examined their biogenesis requirements. Mutations in *SGS3*, *RDR1*, *RDR6*, *DCL2*, and *DCL4* were introduced into *hot3-2* by genetic crosses to generate various double and triple mutants (Fig. 4*A*). Northern blotting with a 600-bp probe corresponding to the 3′ portion of 18S rDNA or with the specific sense probe P1692 (*SI Appendix*, Fig. S1*B*) detected sRNAs in *hot3-2* but not *hot3-2 dcl2-1 dcl4-2* (Fig. 4*B*), indicating that the rRNA-derived sRNAs were indeed DCL2/4-dependent risiRNAs. These risiRNAs, however, were still present in *hot3-2 sgs3-14* and *hot3-2 rdr6-15*, unlike the *TAS3*-derived siRNA *TAS3*_5′D7(+) that was missing in these mutants (Fig. 4*C*), indicating that 18S-risiRNA biogenesis in *hot3* was SGS3 and RDR6 independent. By contrast, the 18S-risiRNA detected by P1692 (18S-P1692) was gone in *hot3-2 rdr1-1* (Fig. 4*D*) and *hot3-2 rdr1-1 rdr6-15* (Fig. 4*C*), revealing that RDR1 was required for 18S-risiRNA biogenesis in *hot3-2*.

### risiRNAs Are Mainly in Nonribosome Fractions and Do Not Affect 18S rRNA Maturation.

**Fig. 5.**
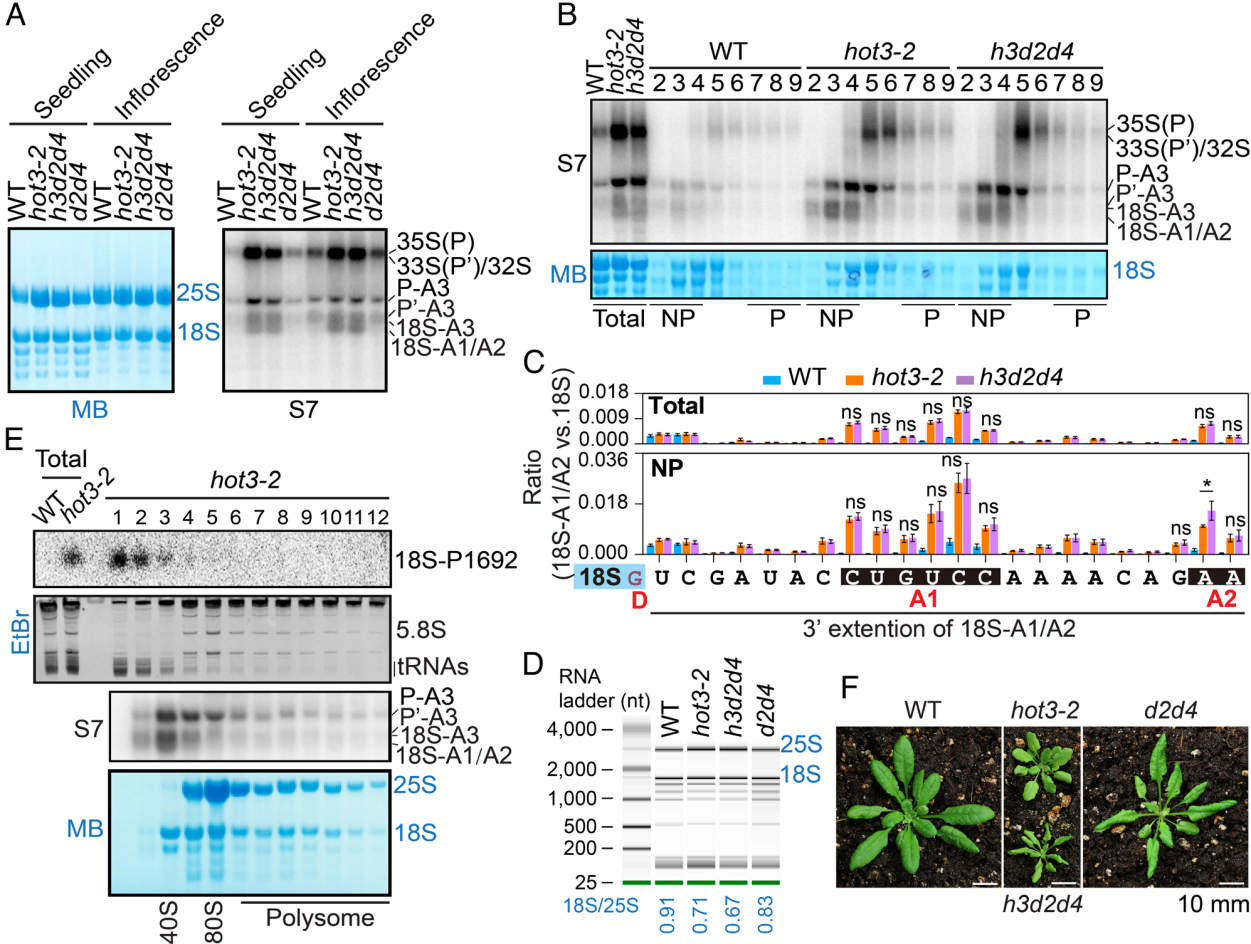
risiRNAs in *hot3* are predominantly in ribosome-free fractions and do not affect 18S rRNA maturation. (*A*) Pre-rRNA northern blotting with the probe S7 for total RNAs from seedlings and inflorescences of WT, *hot3-2, hot3-2 dcl2-1 dcl4-2* (*h3d2d4*), and *dcl2-1 dcl4-2* (*d2d4*). The methylene blue (MB)–stained membrane is shown on the *Left* to indicate relative loading. (*B*) Distribution of pre-18S processing intermediates in sucrose gradient fractions from WT, *hot3-2*, and *hot3-2 dcl2-1 dcl4-2* (*h3d2d4*) seedlings. NP, nonpolysome fractions; P, polysome fractions; Total, total lysate before fractionation. Probe S7 was used. The methylene blue (MB)–stained membrane is shown below. (*C*) Abundance of pre-18S rRNAs ending at each nucleotide beyond the 3′ end of mature 18S rRNA in Total and nonpolysome (NP) fractions from WT, *hot3-2*, and *hot3-2 dcl2-1 dcl4-2* (*h3d2d4*) based on 18S-ENDseq. The abundance is relative to that of mature 18S rRNA ending at the G residue in the blue rectangle. Letters D, A1, and A2 are major processing sites. Mean values of four replicates with SD are shown. The unpaired nonparametric *t* test was used for the evaluation of significance of differences between *hot3-2* and *hot3-2 dcl2-1 dcl4-2*. ns: no significant difference. *: 0.01 ≤ *P* < 0.05. (*D*) Bioanalyzer analysis for seedling total RNAs of WT, *hot3-2, hot3-2 dcl2-1 dcl4-2* (*h3d2d4*), and *dcl2-1 dcl4-2* (*d2d4*). The ratio of 18S:25S rRNA is shown below. (*E*) Distribution of the 18S-risiRNA detected by probe P1692 and pre-rRNAs detected by S7 in sucrose gradient fractions of WT and *hot3-2*. The image of ethidium bromide (EtBr)–stained gel before RNA membrane transfer is shown to indicate relative loading for the P1692 blot. The methylene blue (MB)–stained membrane indicates relative loading for the S7 blot. The positions of 40S SSU, 80S monosome, and polysomes are estimated based on the distributions of 18S, 5.8S, and 25S rRNAs. (*F*) 40-d-old seedlings of WT, *hot3-2, hot3-2 dcl2-1 dcl4-2* (*h3d2d4*), and *dcl2-1 dcl4-2* (*d2d4*) grown in soil (Scale bars, 10 mm).

To evaluate whether risiRNAs in *hot3* exert any functions in 18S rRNA maturation or metabolism, we employed northern blotting to detect and compare pre-18S rRNA processing intermediates in related genetic mutants with (*hot3-2*) or without (*hot3-2 dcl2-1 dcl4-2*) risiRNAs. We found that blocking risiRNA biogenesis in *hot3-2 dcl2-1 dcl4-2* did not rescue aberrant pre-18S rRNA processing in *hot3-2*, as evidenced by similar levels of 18S-A1/A2 (Fig. 5*A*). Like in *hot3-2*, the aberrantly accumulated 18S-A1/A2 rRNAs in *hot3-2 dcl2-1 dcl4-2* were present largely in the nonpolysome fractions (Fig. 5*B*). We also performed 18S-ENDseq to explore, at single-nucleotide resolution, whether risiRNAs affected the 3′ end maturation of 18S rRNA. 18S-ENDseq was done with total RNA and nonpolysomal RNA from wild type, *hot3-2*, and *hot3-2 dcl2-1 dcl4-2*, each with four biological replicates. The abundances of 18S-A1/A2 species in *hot3-2 dcl2-1 dcl4-2* were comparable to those in *hot3-2* (Fig. 5*C*). It indicated that risiRNAs were not responsible for the aberrant 18S rRNA 3′ end processing observed in *hot3*. Accordingly, the lower 18S:25S rRNA ratio in *hot3* was not recovered in *hot3-2 dcl2-1 dcl4-2* (Fig. 5*D*). Like *hot3-2 dcl2-1 dcl4-*2, blocking risiRNAs in *hot3-2 rdr1-1* did not rescue aberrant pre-18S rRNA processing (*SI Appendix*, Fig. S7*A*) or the lower ratio of 18S:25S rRNAs in *hot3-2* (*SI Appendix*, Fig. S7*B*). Interestingly, the 18S-risiRNA detected by P1692 was mainly localized in the ribosome-free fractions 1 and 2 in sucrose gradient sedimentation experiments rather than fraction 3 consisting of 40S SSU, where 18S-A1/A2 intermediates were enriched (Fig. 5*E*).

The evidence presented above indicated that risiRNAs do not affect 18S rRNA maturation in *hot3*. Consistent with this conclusion, blocking risiRNA accumulation in *hot3-2 rdr1-1* and *hot3-2 dcl2-1 dcl4-2* did not alleviate the developmental phenotypes of *hot3-2* (Figs. 4*A* and 5*F*). For example, *hot3-2 dcl2-1 dcl4-2* displayed an additive phenotype of small size like *hot3-2* and downwardly curved leaves as in *dcl2-1 dcl4-2* (Fig. 5*F*).

### Genome-Wide Ribosome Stalling at the ATG Start Codon in *hot3* Mutants.

HOT3/eIF5B1 is a conserved translation initiation factor (37), but its genome-wide function in translation has never been examined at the single-nucleotide resolution in any species. The presence of risiRNAs in *hot3* mutants also raised the question whether risiRNAs affect the functions of HOT3 in mRNA translation initiation. As *hot3* mutants exhibited delayed germination and slow growth at the seedling stage (37), we chose inflorescences from WT, *hot3-2*, *hot3-2 dcl2-1 dcl4-2*, and *dcl2-1 dcl4-2* for ribosome profiling. Independent ribo-seq replicates showed high reproducibility (*SI Appendix*, Fig. S8*A*). The ribosome-protected footprints (RPFs) in all libraries peaked at 28 nt (*SI Appendix*, Fig. S8*B*), like the canonical RPF length observed in plants (64, 65). Most importantly, the RPFs displayed a strong 3-nt periodicity (*SI Appendix*, Fig. S8*C*), suggesting that the ribo-seq datasets were of high quality.

**Fig. 6.**
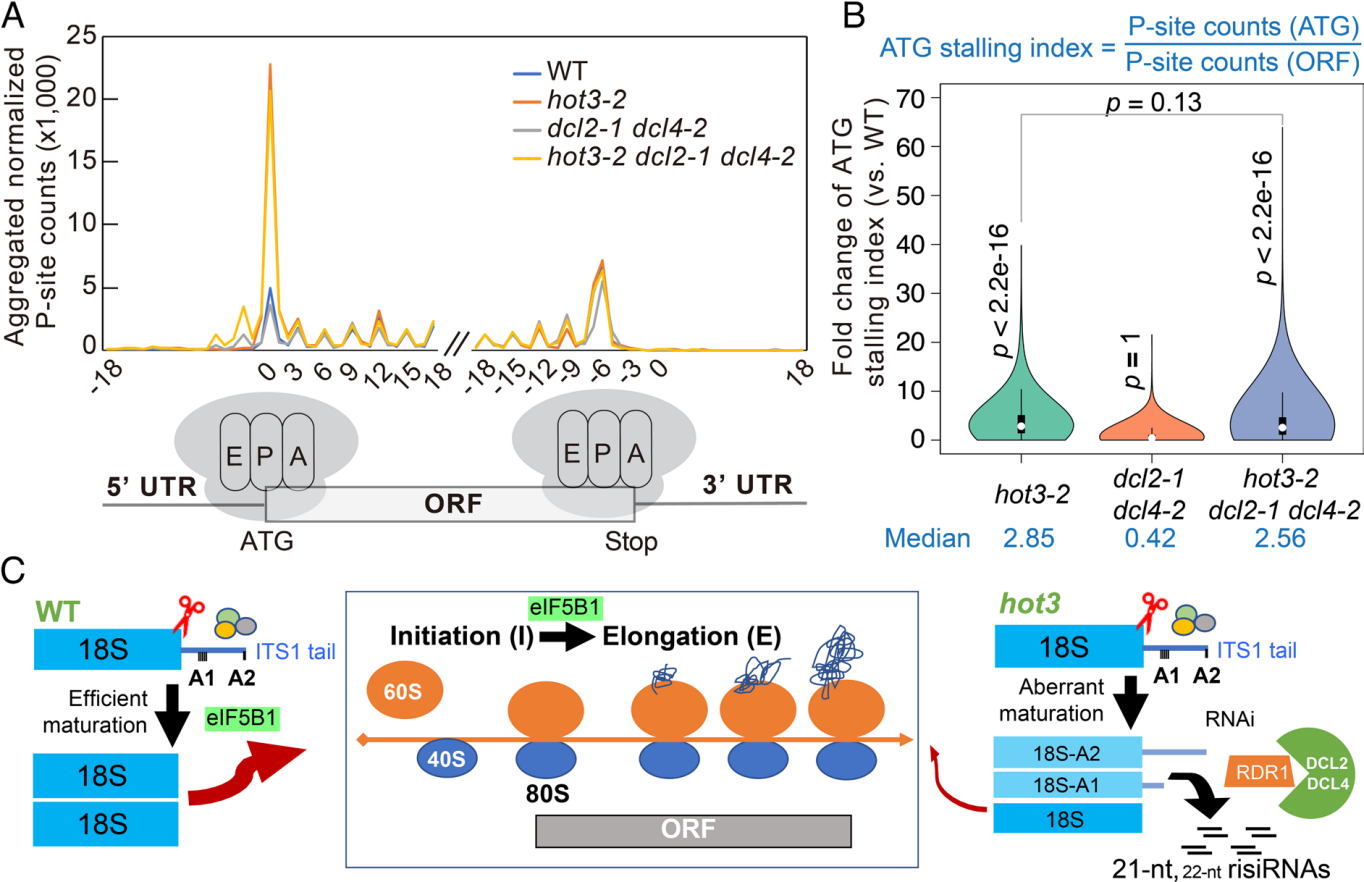
Genome-wide ribosome stalling frequently occurs at the ATG start codon in *hot3.* (*A*) Metagene analysis of aggregated normalized P site counts mapped to regions near the ATG start codon (translation initiation site) and stop codon of annotated ORFs. A 41-nt region near the ATG start codon and stop codon is shown. The diagrams for A site (the entry point for aminoacyl-tRNA), P site (where the peptide bond formation occurs), and E site (the exit site of the uncharged tRNA) within ribosomes are shown below. (*B*) Violin plots for fold change of the ATG stalling index for coding genes in *hot3-2*, *hot3-2 dcl2-1 dcl4-2*, and *dcl2-1 dcl4-2* as compared to WT. The ATG stalling index represents the degree of ribosome stalling on the ATG and is determined by calculating the ratio of P site counts on the ATG start codon of one annotated gene versus all P site counts mapped to ORF. The numbers in blue font at the *Bottom* indicate the median values of each genotype. The single-tailed *t* test was used for evaluation of significant differences between each genotype and WT, with the *P* value shown vertically. *P* < 0.0001 (****). The *P* value for the difference between *hot3-2* and *hot3-2 dcl2-1 dcl4-2* is shown horizontally. (*C*) Model of HOT3 coordinating late 40S maturation, translation initiation (I)–elongation (E) transition, and RNAi in *Arabidopsis*. Late 40S maturation coupled with 18S rRNA biogenesis is coordinated with mRNA translation initiation. In WT, 18S rRNA 3′ end maturation includes the 3′ to 5′ exonucleolytic processing from A2 to A1 hotspots by ribosome biogenesis factors (ovals) and AtNOB1 (scissor)-mediated complete removal of ITS1. HOT3/eIF5B1 facilitates these late 40S assembly events, thereby allowing translation-competent 80S ribosomes to undergo the transition from initiation to elongation. Dysfunction of HOT3 causes aberrant 18S rRNA maturation in 40S SSU biogenesis and constrains the formation of translation-competent 80S ribosomes, resulting in genome-wide ribosome stalling at the ATG start codon and reduced I–E transition. On the other hand, aberrant pre-rRNA processing or degradation triggers RNAi, resulting in the production of RDR1- and DCL2/4-dependent risiRNAs. risiRNAs in *hot3* are mainly distributed in ribosome-free fractions and barely affect ribosome biogenesis and genome-wide translation initiation.

To identify and quantify the codons being translated, we subjected our datasets to RiboTaper analysis (66), which defines translated ORFs based on the 3-nt periodicity of peptidyl site (P site) signals (67). We mapped the P site signals to the *Arabidopsis thaliana* genome and determined the locations of the P site nucleotides. As expected, most P site nucleotides were in the body of coding sequences in all backgrounds (*SI Appendix*, Fig. S8*D*). We further examined the distribution of P sites at all annotated ORFs. Metagene analysis uncovered that a larger number of P sites were found at the ATG start codon in the *hot3-2* mutants (Fig. 6*A*), which was consistent with the function of eIF5B in the transition from translation initiation to elongation as defined by biochemical studies in yeast (21, 31, 32). We developed an ATG stalling index to represent the extent of ribosome stalling at the start codon (Fig. 6*B*). Compared with wild type, the ATG stalling indexes in *hot3-2 dcl2-1 dcl4-2* and *hot3-2* were significantly increased, with median values of 2.56 and 2.85, respectively (Fig. 6*B*). Notably, the *dcl2-1 dcl4-2* mutant did not show any significant difference from wild type in ribosome stalling (Fig. 6*B*). No significant differences in ribosome stalling at the ATG start codon were observed between *hot3-2 dcl2-1 dcl4-2* and *hot3-2* (Fig. 6*B*). Altogether, dysfunction of HOT3 triggered genome-wide ribosome stalling at the ATG start codon independently of risiRNAs.

## Discussion

The final steps in 18S rRNA maturation at the 3′ end have been unclear in plants. By developing and implementing 18S-ENDseq, we uncovered previously unknown events in 18S rRNA 3′ end maturation or metabolism. For example, 18S-A1 species representing processing hotspots between D and A2 sites were quantitatively defined, and adenylation of 18S-A1 and 18S-A2 was uncovered. Although much of the study was focused on *hot3-2*, in which 18S-A1/A2 species as well as their 3′ adenylated variants were increased in abundance, these processing or metabolic intermediates were also present in wild type. 18S species with heterogeneous 3′ extremities upstream of A2 were observed in *Arabidopsis* *rrp6l2* by 3′ RACE (25) and in rice by circular RT-PCR analysis (9), although they were not annotated as 18S-A1. In this study, with the highly quantitative 18S-ENDseq, we showed that the abundance of 18S-A1 species was actually higher than that of 18S-A2 species in wild type (*SI Appendix*, Fig. S5), suggesting that 18S-A1 species are processing or degradation intermediates. Given the heterogeneous ends of 18S-A1 species, we suspect that they are derived from 3′ to 5′ trimming of 18S-A2. The presence of multiple As at the 3′ ends of 18S-A2 is also consistent with 18S-A2 being trimmed from the 3′ end as adenylation is often associated with exosome-mediated 3′ to 5′ trimming in pre-rRNA maturation (48). In fact, nontemplated nucleotide additions were found for 18S rRNA species with 3′ ends between D and A2 sites in *Arabidopsis* *rrp6l2* and rice (9, 25), supporting the notion that plant cells employ progressive 3′ to 5′ exonucleolytic digestion after cleavage at A2 (25), like yeast and human cells (18, 68). We propose that 18S-A1/A2 species are the direct precursors to 18S rRNA, although it is also possible that they are intermediates in pre-rRNA degradation.

This study also uncovered two molecular functions of HOT3. One is that HOT3 is necessary for the 3′ end maturation of 18S rRNA. We found that, relative to mature 18S rRNA, the abundance of 18S-A1/A2 was 1.46% in wild type and reached 6.7% in *hot3-2*, suggesting that HOT3 is required for the removal of the 3′ extension in 18S-A1/A2 to produce the mature 18S rRNA. The decreased processing efficiency in *hot3* may lead to the lower 18S:25S rRNA ratio. Another function of HOT3 is to promote the transition from translation initiation to elongation, as evidenced by a global stalling of 80S ribosomes at the ATG start codon in *hot3-2* (Fig. 6*A*). In yeast, the physical interaction between pre-40S and 60S LSU triggers the GTPase activity of eIF5B/Fun12 to induce a structural rearrangement within pre-40S to allow NOB1 to cleave the extended tail of 18S (22). In light of the biochemical insights from yeast and our molecular and genomic results, we propose that HOT3 in *Arabidopsis* also works at the final stage in translation initiation to enable 18S rRNA maturation in 80S ribosomes. However, given that 18S-A1/A2 mainly resides in the 40S fraction, it is also possible that HOT3 acts in the 40S SSU to promote 18S rRNA 3′ end maturation.

We show that HOT3 also constrains RDR1- and DCL2/4-dependent risiRNA biogenesis. In *Arabidopsis*, risiRNA biogenesis has been reported to occur in two mutants with defects in pre-rRNA processing (17, 69), one being a null mutant of the nuclear exosome cofactor AtMTR4 and the other being *fry1*. A common feature among *mtr4*, *fry1*, and *hot3* mutants is that the risiRNAs are DCL2 and DCL4 dependent (17, 69). Interestingly, we note some differences in risiRNA biogenesis in the three mutants: risiRNAs are derived from the 5′ ETS P-P′ region in *mtr4* (69), from the transcribed spacers including 5′ ETS, ITS1, ITS2, and 3′ ETS in *fry1* (17), and mainly from the 3′ portion of 18S rRNA in *hot3* (this study). The locations of risiRNAs along the pre-rRNA largely correlate with where the proteins act in pre-rRNA metabolism. For example, during early processing in the nucleus, the 5′ ETS is probably degraded by the nuclear exosome. In *mtr4*, the accumulation of the 5′ ETS P-P′ fragment could trigger RNAi (69). In *fry1*, the activities of nuclear XRN2 and XRN3 are compromised (17). These 5′ to 3′ exonucleases probably help to degrade the transcribed spacers after processing or trim these spacers during processing (43, 69). Their abnormal accumulation in *fry1* probably triggers RNAi. In *hot3*, the processing defect lies at the 3′ end of 18S rRNA, consistent with risiRNA accumulation in this region. Another difference is that risiRNA biogenesis requires RDR6 in *fry1* but RDR1 in *hot3*. We suspect that this has to do with the subcellular sites of risiRNA biogenesis. In *fry1*, the aberrant pre-rRNA processing events are early in the pathway, and the nontranscribed spacers probably never escape the nucleus. In *hot3*, the aberrant 18S-A1/A2 processing is at the late 40S maturation stage coupled with mRNA translation initiation, and thus, 18S-A1/A2 may reside in the cytoplasm. The different subcellular compartments of the precursor RNAs to risiRNAs may have dictated the RDR6 vs. RDR1 requirement. Consistent with this hypothesis, in *fry1*, coding gene–derived (i.e., mRNA-derived) siRNAs are RDR1 dependent, suggesting that RDR1 acts in the cytoplasm. It is also worth noting that viral infection can trigger the production of risiRNAs (a type of vasiRNA) in an RDR1-dependent manner (63, 70). It is possible that viral infection impacts pre-rRNA processing or rRNA metabolism to result in aberrant rRNA. Alternatively, RDR1, which is highly induced upon viral infection (71), can access abundant RNAs such as rRNAs.

Blocking risiRNA accumulation in *hot3-2 rdr1-1* or *hot3-2 dcl2-1 dcl4-2* did not rescue defects in morphology or 18S rRNA maturation in *hot3-2.* Similarly, the *mtr4 dcl2 dcl4* triple mutant did not rescue the *mtr4* single mutant in either morphological or rRNA metabolism defects (69). This indicates that risiRNAs in *mtr4* and *hot3* are by-products of rRNA metabolism without many effects on either plant morphology or rRNA maturation. For *fry1 rdr6* and *fry1 dcl2 dcl4*, blocking risiRNA biogenesis released the competition between risiRNAs and miRNAs for AGO1 occupancy and partially alleviated the leaf phenotype of *fry1* (17). We note that the abundance of risiRNAs is much higher in *fry1* (17) than in *hot3*, which could explain the lack of an inhibitory effect of risiRNAs on miRNAs in *hot3*. However, it remains possible that risiRNAs in *hot3* affect specific miRNAs or other siRNAs through competition for AGO1. It is also possible that risiRNAs can target other RNAs themselves.

In summary, our studies show that HOT3 coordinates 18S rRNA maturation and mRNA translation initiation in *Arabidopsis thaliana*, and this molecular function of HOT3 also suppresses RNAi of rRNAs (Fig. 6*C*). In WT under physiological conditions, upon endonucleolytic cleavage at the A2 site, 18S-A2 intermediates probably undergo 3′ to 5′ exonucleolytic processing to trim the 3′ ends to A1 hotspots. HOT3 may facilitate AtNOB1-mediated cleavage of 18S-A1/A2 species at the D site to remove the 3′ extensions in ITS1 for 18S rRNA maturation, thereby allowing 40S SSU to form translation-competent 80S ribosomes. On the other hand, and consistent with biochemical studies in yeast, our genome-wide ribosome profiling revealed a role of HOT3 in the transition from translation initiation to elongation. Therefore, it is likely that HOT3 coordinates 18S rRNA maturation and the final stage of translation initiation. Dysfunction of HOT3 causes aberrant 18S rRNA maturation in 40S SSU biogenesis and constrains the formation of translation-competent 80S ribosomes, leading to increased accumulation of 18S-A1/A2 and reduced levels of mature 18S rRNA. Dysfunction of HOT3 also causes genome-wide ribosome stalling at the ATG start codon during mRNA translation initiation. The aberrant rRNA maturation probably triggers RNAi to produce risiRNAs (Fig. 6*C*).

## Materials and Methods

### Plant Materials.

*Arabidopsis thaliana* mutants and transgenic lines used in this study are in the Columbia (Col-0) ecotype. The *hot3-2* allele (SALK_124251) was shared by Elizabeth Vierling and Huiru Peng (37). Further details can be found in *SI Appendix*, *Materials and Methods*.

### 18S-ENDseq Library Construction and Analysis.

The 18S-ENDseq library construction pipeline was adapted from the circular RT-PCR assay (40, 41) with modifications. RNA self-ligation was followed by reverse transcription with the RT primer 18c2 using the RevertAid First Strand cDNA Synthesis Kit (Thermo Fisher, K1621). A custom-made universal primer (Uni-r5) containing the Illumina P5 sequence and 12 indexed Illumina P7 primers in *SI Appendix*, Table S1 were then employed to amplify 18S-ENDseq libraries. PCR products were size-selected, and the barcoded libraries were pooled for pair-end 150-bp sequencing in an Illumina HiSeq 2500 machine. Further details can be found in *SI Appendix*, *Materials and Methods*.

### sRNA Sequencing and risiRNA Analysis.

Here, 20 μg total RNA was resolved on a 15% urea polyacrylamide gel, and sRNAs of 18 to 40 nucleotides (nt) were recovered from excised gel pieces corresponding to RNAs of this size range. Small RNA libraries were constructed following instructions from the NEBNext Multiplex Small RNA Library Prep Set for Illumina (E7300). The resulting data were analyzed by a homemade pipeline pRNASeqTools v0.8 (https://github.com/grubbybio/pRNASeqTools). Further details can be found in *SI Appendix*, *Materials and Methods*.

### RNA-seq and Ribo-seq Library Construction.

Polyadenylated RNA isolated from total RNA using the NEBNext Poly(A) mRNA Magnetic Isolation Module (NEB, E7490L) was subjected to RNA-seq library construction with the NEBNext Ultra Directional RNA Library Prep Kit for Illumina (NEB, E7420L). Ribosome profiling libraries were generated based on a published protocol (64) with some modifications. Further details can be found in *SI Appendix*, *Materials and Methods*.

Additional methods are available in *SI Appendix*, *Materials and Methods*.

## Supplementary Material

Appendix 01 (PDF)

## Data Availability

All sequencing data have been deposited in the Gene Expression Omnibus under the accession GSE223172 (72). All study data are included in the article and/or *SI Appendix*.
