# Supplementary material for: Arabidopsis HOT3/eIF5B1 constrains rRNA RNAi by facilitating 18S rRNA maturation
Source: Proc Natl Acad Sci U S A. 2023 Apr 3;120(15):e2301081120. doi: 10.1073/pnas.2301081120 (PMC10104536; doi:10.1073/pnas.2301081120)
Supplement: Supplementary file 1 — Appendix 01 (PDF) [file pnas.2301081120.sapp.pdf]

**Supporting Information for**
# Arabidopsis HOT3/eIF5B1 constrains rRNA RNAi by facilitating 18S rRNA maturation

Runlai Hang, Ye Xu, Xufeng Wang, Hao Hu, Nora Flynn, Chenjiang You, and Xuemei Chen

Corresponding authors:

Xuemei Chen
Email: xuemei.chen@ucr.edu; xuemei.chen@pku.edu.cn

Chenjiang You
Email: cjyou@scau.edu.cn

**This PDF file includes:**

Extended Materials and Methods
Figures S1 to S8
Tables S1
SI References

## Extended Materials and Methods

### Plant materials and growth conditions

Arabidopsis mutants and transgenic lines used in this study are in the Columbia (Col-0) ecotype. The *hot3-2* allele (SALK_124251) was shared by Dr. Elizabeth Vierling and Dr. Huiru Peng (1). The T-DNA insertion mutants of *hot3-3* (SAIL_1147_G06) and *sgs3-14* (SALK_001394) were from the Arabidopsis Biological Resource Center. The double mutants of *rdr1-1* (SAIL_672F11) *rdr6-15* (SAIL_617_H07) and *dcl2-1* (SALK_064627) *dcl4-2* (GABI_160G05) were as described (2). Additional mutants including *hot3-2 sgs3-14*, *hot3-2 rdr6*-15, *hot3-2 rdr1-1*, *hot3-2 dcl2-1 dcl4-2*, and *hot3-2 rdr1-1 rdr6-15* were generated by genetic crosses. Mutants were confirmed by genotyping using primers as shown in Supplemental Table S1. Plants were grown in soil in growth rooms or on 1⁄2 Murashige & Skoog Modified Basal Salt Mixture (PhytoTech Labs, M524) plus 1% Sucrose (Fisher Scientific, BP220-10) and 0.8% Agar (Sigma-Aldrich, A1296) in growth chambers at 22°C under full-spectrum white fluorescent light under long-day (16 h light/8 h dark) conditions. Inflorescence or 18-day-old seedlings were collected, frozen in liquid nitrogen, and stored in -80°C until used.

### Generation of transgenic complementation lines of *HOT3::HOT3-EYFP* in the *hot3-2* background

A 6.416-kbp genomic fragment of *HOT3* (AT1G76810), corresponding to the region from base 28,667,024 to 28,673,439 of Arabidopsis chromosome 1 annotated in National Center for Biotechnology Information, was amplified by HOT3g_FP and HOT3g_RP (Table S1). The DNA fragment containing the *HOT3* promoter plus 5' UTR (1.474kb) and coding region without the stop codon was subcloned into TSK108 at EcoRI and BamHI sites by using NEBuilder® HiFi DNA Assembly Master Mix (NEB, E2621L). After confirmation by DNA sequencing, the *HOT3* genomic DNA (HOT3g) was cloned into the destination vector pGWB640 using Gateway LR Clonase II Enzyme mix (Thermo Fisher, 11791100) to generate pGWB640-HOT3g, in which *HOT3* is fused to EYFP and driven by the *HOT3* promoter. After the plasmid was introduced into the *Agrobacterium tumefaciens* strain GV3101, transformation was done using the floral dipping method (3) in the *hot3-2* background. *HOT3::HOT3-EYFP hot3-2* transgenic lines were selected in soil by Basta (Glufosinate Ammonium). Transgenic lines were confirmed by fluorescence py detection for EYFP signals.

### Determination of HOT3's subcellular localization

A 3.954-kb genomic fragment of the nucleolar marker Fibrillarin 2 (FIB2) (At4g25630), comprising 1.99-kb promoter + 5' UTR and 1.964-kb gene body region, was amplified by FIB2g_FP and FIB2g_RP and cloned into TSK108 at EcoRI and BamHI sites by using the NEBuilder® HiFi DNA Assembly Master Mix (NEB, E2621L). After DNA sequencing, FIB2g was subcloned into pGWB5017, which was reported before (4), to generate the destination construct pGWB5017-FIB2g, in which *FIB2* is fused to *mRuby* and driven by the *FIB2* promoter. After the plasmid was introduced into the *Agrobacterium tumefaciens* strain GV3101, plant transformation was performed with *HOT3::HOT3-EYFP hot3-2* plants homozygous for both *HOT3::HOT3-EYFP* and *hot3-2*. The 10-day-old T1 lines of *FIB2::FIB2g-mRuby* in the *HOT3*::*HOT3-EYFP hot3-2* background were selected by 25 µg/mL hygromycin B (Roche, 10843555001) on MS medium, and stained with 10 µg/mL propidium iodide (PI) and 1 mg/ml DAPI for 1 min. PI/mRuby and EYFP were visualized using wavelengths of 600–640 nm and 500–540 nm, respectively. Images were taken with a Zeiss LSM510 inverted confocal microscope.

### Northern blot analysis for rRNAs and small RNAs

Total RNA was isolated from 18-day-old seedlings using TRI Reagent (MRC, TR118) according to the manufacturer's instructions. Three micrograms of total RNA were separated on a 1.2% (wt/vol) agarose/formaldehyde gel and transferred to an Hybond N+ hybridization membrane (Cytiva, RPN303B) by capillary elution, for rRNA detection (5, 6). Five or ten micrograms of total RNA were separated on a 15% (wt/vol) acrylamide/7 M urea gel and transferred to an Hybond NX membrane (Cytiva, RPN303T) by electrotransfer for small RNA detection. The 5'-end labeling of short DNA oligonucleotides was performed with γ-32P-ATP (PerkinElmer, NEG502A001MC) and T4 polynucleotide kinase (NEB, M0201). The labeling of longer DNA fragments was performed with $\alpha$-32P-dCTP (PerkinElmer, NEG513H500UC) and Random Primer DNA Labeling Kit Ver.2.0 (TAKARA, 6045). Hybridization was performed overnight in Church buffer (7) at 55°C for rRNA and 45°C for sRNA. Membranes were washed and exposed to a phosphor screen (GE Healthcare), and radioisotope signals were detected by a Typhoon TRIO scanner (GE Healthcare). A list of primers is provided in Supplemental Table S1.

**Polyribosome isolation and sucrose gradient fractionation**

Polyribosome isolation and fractionation were performed according to the method described before (8, 9), with minor modifications. Approximately 0.25g of frozen seedling or inflorescence was ground into fine powder in liquid nitrogen. One milliliter of polysome extraction buffer (PEB) (0.2M Tris-HCl pH8.0, 0.2M KCl, 35mM $MgCl_2$, 25mM EGTA, 1% TritonX-100, 2% polyoxyethylene-10-tridecyl ether (PTE), 100mM 2-Mercaptoethanol, 25ug/ml cycloheximide (CHX), 100ug/ml chloramphenicol (CHL) and 0.5mg/ml heparin) was added and mixed thoroughly with the powder. After 10 minutes incubation on ice, cell debris was removed by centrifugation for 5 min at 12,000 rpm in a microcentrifuge at 4 °C. Sodium deoxycholate (DOC) was added to the supernatant to a final concentration of 0.5%, and the lysate was kept on ice for 5 min. After centrifugation for 15 min at 12,000 rpm in a microcentrifuge, the supernatant was transferred to a new 1.5ml RNase-free tube and marked as PEB-input. 0.5 ml of the PEB-input was loaded onto a 4.4-ml sucrose gradient (15-55% Sucrose in 40mM Tris-HCl pH8.0, 20mM KCl, 10mM $MgCl_2$, 25ug/ml CHX, 100ug/ml CHL, 0.5mg/ml heparin). After ultracentrifugation in a Beckman SW-55Ti rotor at 45,000 rpm for 65 minutes at 4°C, density gradient fractionation (Brandel, SYN-202) was performed as described before (10, 11). The fluid speed was 1.5 ml/min, and collection time for each fraction was 19 seconds. Ethylenediamine tetraacetic acid (EDTA) and sodium dodecyl sulfate (SDS) were added to 20mM and 0.5%, respectively, into each fraction at room temperature. RNA was immediately extracted from each fraction using TRI-reagent (MRC, TR118) according to the manufacturer's instructions. Pelleted RNA was resuspended in 25ul RNase-free ddH2O for library construction or TE buffer (10mM Tris-HCl pH7.5, 1mM EDTA) for northern blot assays. 5ul (20%) of RNA in each fraction was used in northern blot assays for rRNAs or risiRNAs.

**Circular RT-PCR**

The circular RT–PCR assay was performed as described previously (5, 12) with minor modifications. 1.5ug of total RNA was self-ligated into circular RNA by 30 units T4 RNA ligase 1 (New England Biolabs, M0437M) in the reaction mixture containing 10 mM ATP and 40 units RiboLock RNase Inhibitor (Thermo Fisher, EO0381) in thermomixer at 37 °C for 2 h. Circularized RNA was recovered by Phenol-chloroform extraction and ethanol precipitation in the presence of 0.3 M sodium acetate (pH 5.2) and 1ug glycogen (Thermo Fisher, R0551). Then RevertAid First Strand cDNA Synthesis Kit (Thermo Fisher, K1621) was used for the reverse transcription step using 2 pmol of either 18c2 or 25c1 as the RT primer. PCR reactions were done using OneTaq® Quick-Load® 2X Master Mix with Standard Buffer (New England Biolabs, M0486L) for 14 cycles. DNA products were resolved in a 1% agarose gel and bands, bands were excised from the gel, and the DNA was recovered by Zymoclean Gel DNA Recovery Kit (Zymo, D4008) and cloned in the pJET1.2/blunt vector by CloneJET PCR cloning kit (Thermo Fisher, K1232). Positive clones were further verified by colony PCR using pJET1.2 forward and reverse sequencing primers. Candidate clones were subjected to sanger sequencing, using the pJET1.2 sequencing primer. DNA sequencing data were analyzed by combining Basic Local Alignment Search Tool (BLAST)

from the National Center for Biotechnology Information (NCBI) and the home-made 45S rDNA reference (13). A complete list of primers is provided in Supplemental Table S1.

**18S-ENDseq library construction and analysis**

The 18S-ENDseq library construction pipeline was adapted from the circular RT–PCR assay with modifications. 1.5ug of RNA was used for RNA self-ligation followed by reverse-transcription with the RT-primer 18c2, using the RevertAid First Strand cDNA Synthesis Kit (Thermo Fisher, K1621). A custom-made universal primer (Uni-r5) containing the Illumina P5 sequence and 12 indexed Illumina P7 primers in Table S1 were then employed to amplify 18S-END libraries, using the SYBR-Green qPCR Supermix (BioRad, 170-8882) in a CFX96 Real-Time system (BioRad, C1000 Touch) for 15 cycles. PCR products were size selected in an agarose gel and recovered by QIAEX II Gel Extraction Kit (150) (QIAGEN,20021), or directly purified with AMPure XP Beads (Beckman, A63881). After quality control and quantification with a Bioanalyzer (Agilent, 2100), the barcoded libraries were pooled for pair-end 150-bp sequencing in an Illumina Hiseq 2500 machine. A list of oligonucleotides is provided in Supplemental Table S1. For non-polysomal and polysomal RNAs, heparin was replaced by SUPERase•In™ RNase Inhibitor (Thermo Fisher, AM2694) during polysome isolation and fractionation. The final concentrations of the RNase Inhibitor were 40U/ml and 4U/ml in PEB and in the 15%-55% sucrose gradient, respectively. Input RNA was extracted from PEB-input, non-polysomal RNA was extracted from fractions 1 to 4, and polysomal RNA was extracted from fractions 8-12. Resulting reads were first trimmed to remove the 5' adapter (GGATCAACCAGGTA) and then mapped to the 45S rDNA reference that was generated based on earlier reports (13-17) using STAR v2.7.9a with parameters "--outSAMmultNmax 1 --outFilterMultimapNmax 50 --outFilterMismatchNoverLmax 0.1". A homemade Perl script was used to extract, characterize, and count the 3' end of each mapped read.

**sRNA sequencing and risiRNA analysis**

20ug total RNA was resolved on a 15% urea-polyacrylamide gel (Urea-PAGE) and small RNAs of 18~40 nucleotides (nt) were recovered from excised gel pieces corresponding to RNAs of this size range. Small RNA libraries were constructed following instructions from the NEBNext Multiplex Small RNA Library Prep Set for Illumina (E7300). The barcoded libraries were pooled for pair-end 150-bp sequencing in an Illumina Hiseq 2500 instrument. The resulting data were analyzed by a homemade pipeline pRNASeqTools v0.8 (https://github.com/grubbybio/pRNASeqTools). For risiRNA analysis, the raw reads were trimmed to remove the 3' adaptor sequences and filtered based on size (18–42 nt) and quality using cutadapt v4.1. Trimmed reads then were aligned to the 45S rDNA reference (13-17) using ShortStack v3.452 with parameters '-bowtie_m 1000 -ranmax 50 -mmap u -mismatches 1'. Reads corresponding to each rDNA features were split based on strandedness, counted by featureCounts v2.0.3, and normalized to total mapped reads. Differential features with ribosomal siRNAs (fold change ≥ 2 and P value < 0.01) were identified by DESeq2.

**RNA-seq and Ribo-seq library construction**

Total RNA was extracted directly from tissue powder using TRI-reagent (MRC, TR118). Polyadenylated RNA isolated from total RNA using the NEBNext Poly(A) mRNA Magnetic Isolation Module (NEB, E7490L) was subjected to RNA-seq library construction with the NEBNext Ultra Directional RNA Library Prep Kit for Illumina (NEB, E7420L). The barcoded libraries were pooled for pair-end 150-bp sequencing in an Illumina Novaseq 6000. Ribosome profiling libraries were generated based on a published protocol (18) with some modifications as detailed below. 0.2g of inflorescence tissue was ground into fine powder in liquid nitrogen and the powder was lysed in 0.45ml Buffer D, which consists of 100 mM Tris-HCl pH8.0, 40 mM KCl, 20 mM $MgCl_2$, 2% (v/v) polyoxyethylene (10) tridecyl ether (Sigma-Aldrich, P2393), 0.5% (w/v) sodium deoxycholate (Sigma-Aldrich, D6750), 1mM dithiothreitol (Thermo-Fisher, R0861), 100 ug/ml cycloheximide (Sigma-Aldrich, C1988) and 10 U/ml DNase I (Roche, 4716728001). After 10 minutes incubation on ice, two rounds of centrifugation at 14,000 rpm for 10 minutes at 4°C were applied to generate

cleared supernatant marked as total lysate. The $OD_{260}$ of the cleared total lysate was measured by a NanoDrop spectrophotometer (Thermo Scientific, ND-2000), using Buffer D as the blank. Around 2,000U $OD_{260}$ of the total lysate was treated with 75U RNase I (Thermo Fisher, AM2294) at 23°C for 1 hour. The reaction was stopped by adding 300U SUPERase•In RNase Inhibitor (Thermo Fisher, AM2694) and further processed with Amersham MicroSpin S-400 HR columns (Cytiva, 27514001). Ribosome protected footprints (RPFs) were isolated and purified using the RNA Clean & Concentrator-5 kit (Zymo Research, R1016). Later, rRNA depletion was performed as described before (19), using Dynabeads MyOne Streptavidin C1 (Thermo Fisher, 65001) and custom-made biotinylated DNA oligonucleotides listed in Table S1. The rRNA-depleted RPFs were concentrated by the RNA Clean & Concentrator-5 kit and fragments of 20-nt to 40-nt were selected from a 15% urea-PAGE gel. Sliced gel pieces were ground by RNase-free 1ml Olympus Premium Reach Pipet Tips (Genesee Scientific, 23-165RL). Extraction buffer (0.5M ammonium acetate, 10% SDS) was added to extract RPFs at room temperature for 2 hours. Gel debris was removed by Corning Costar Spin-X centrifuge tube filters (Sigma-Aldrich, CLS9301). RPFs were precipitated by incubation with 1ul glycogen (Thermo Fisher, R0551) and 3 volumes of 100% ethanol at -20°C overnight. End repair was performed for RPFs using T4 PNK without ATP for 30 minutes at 37°C, followed by 5' phosphorylation with ATP for additional 30 minutes at 37°C. RPFs recovered from the reactions were dissolved in 3ul RNase-free ddH2O and subjected to small RNA library construction using NEBNext Multiplex Small RNA Library Prep Set for Illumina (NEB, E7300). The barcoded libraries were pooled for pair-end 150-bp sequencing in an Illumina Hiseq X instrument.

### RNA-seq and Ribo-seq reads processing

RNA-seq and Ribo-seq reads were processed with the pRNASeqTools pipeline (https://github.com/grubbybio/pRNASeqTools). Briefly, for RNA-seq, paired raw reads were trimmed with cutadapt v4.1 (20), and then mapped to the Arabidopsis genome TAIR10 (https://www.arabidopsis.org) by STAR v2.7.9a (21) with parameters "--outSAMmultNmax 1 --outFilterMultimapNmax 50 --outFilterMismatchNoverLmax 0.1". Only properly and uniquely mapped read pairs were counted by featureCounts v2.0.3 (22) for downstream analyses. For Ribo-seq, raw reads were also trimmed by cutadapt, and 18-42 nt reads were mapped by STAR.

### P-site and ORF identification

RPFs were processed using the RiboTaper pipeline (23). Briefly, metaplots for all RPFs from 6 samples around the start and stop codons were created by 'create_metaplots.bash', and the P-site offset for RPFs in each length was determined accordingly. Next, 'Ribotaper.sh' was called to identify ORFs with options '26,27,28,29 10,11,12,13'.

### ATG stalling index calculation

The number of P-sites mapped to the ORF (ORF) of each gene ($N_{ORF}$) was counted using P-sites outputs by RiboTaper. P-sites that coincide with ATG translation initiation sites (ATG) were counted by featureCounts separately to obtain $N_{ATG}$. The ATG stalling index for each gene was calculated using the following equation:

$$ATG\ stalling\ index = \frac{N_{ATG}}{N_{ORF}}$$

## Supplemental Figures

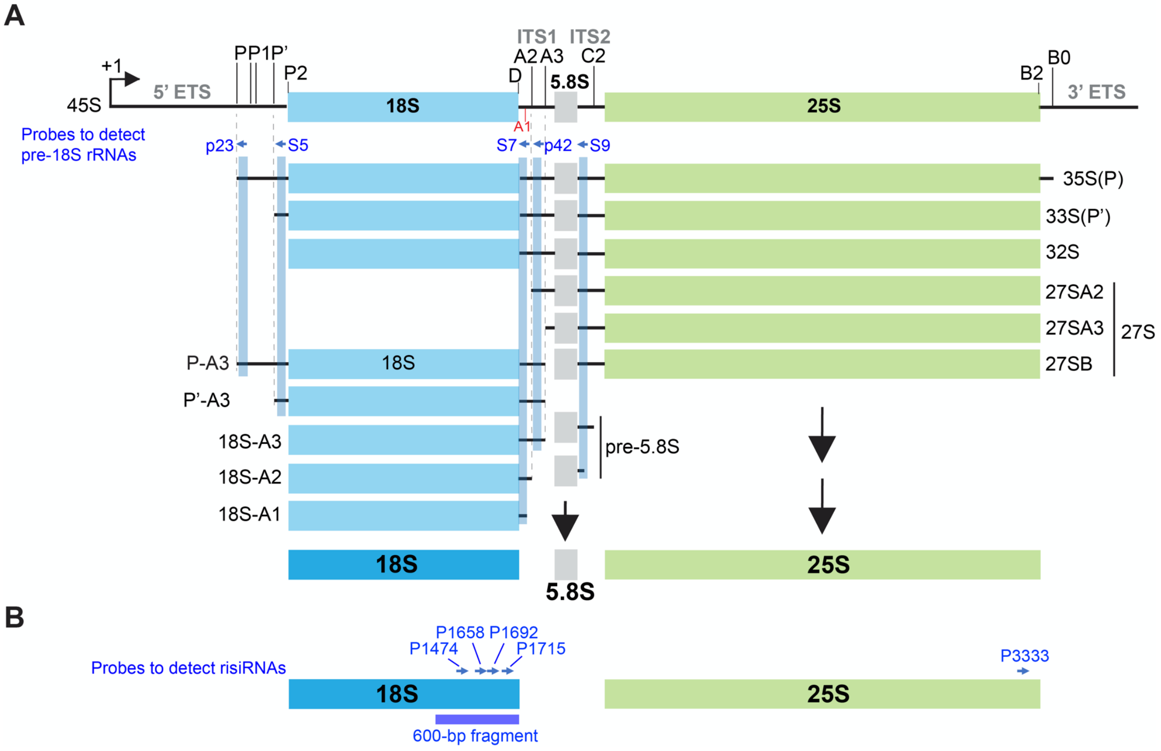


**Fig. S1. Schematic representation of 45S rDNA and the probes used to detect pre-18S rRNAs and risiRNAs*.***

A. 45S rDNA structure, pre-rRNAs, and probes to detect pre-18S rRNA processing intermediates. The blue and green rectangles represent mature 18S and 25S rRNAs, respectively. The full-length pre-rRNA (45S) is shown above with various processing intermediates shown below. +1, transcription start site. The vertical lines above and below the 45S diagram represent processing sites. DNA probes are illustrated in blue with p23 and S5 in 5' ETS, S7 and p42 in ITS1, and S9 in ITS2.

B. DNA probes to detect risiRNAs. For 18S-risiRNAs detection, four DNA oligonucleotides (P1474, P1658, P1692, and P1715 indicated by arrows) and one 600-bp fragment (indicated by the rectangle) are used. The DNA oligonucleotide P3333 is used for 25S-risiRNA detection. The sequences of the DNA oligonucleotides can be found in Table S1. The 600-bp fragment was amplified by PCR using primers R069 and R070 (see Table S1 for their sequences).

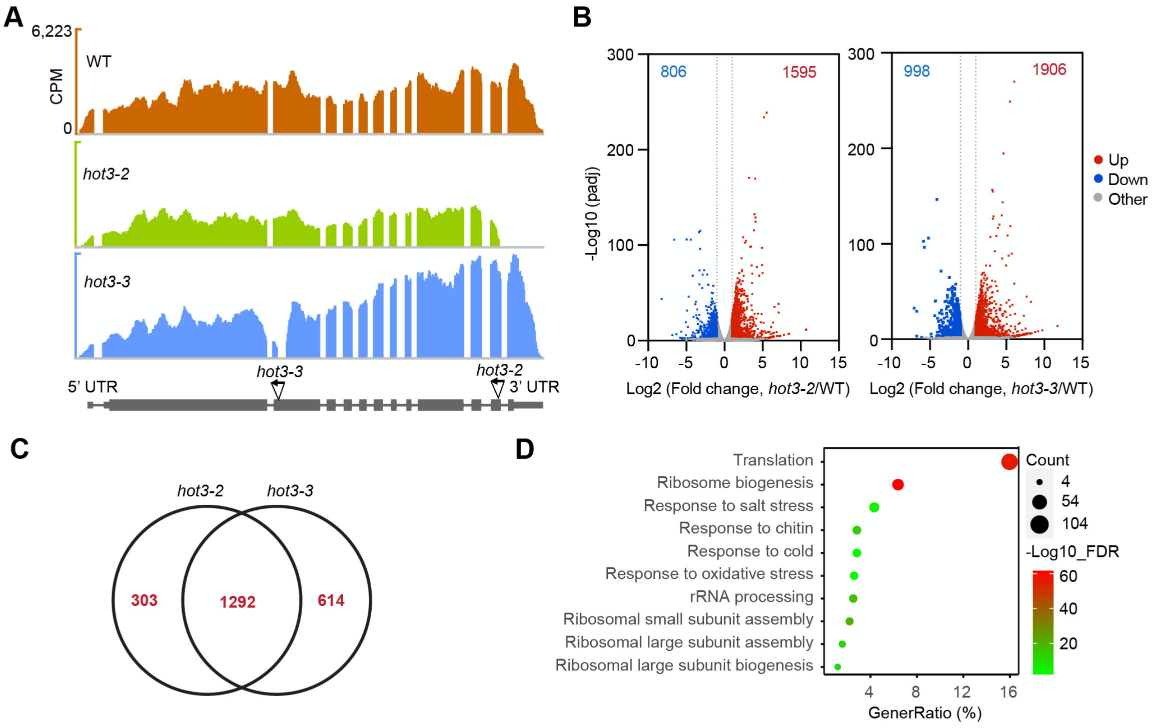


**Fig. S2. Expression of genes in ribosome biogenesis and translation is up regulated in *hot3* mutants.**

A. Genome browser views of *HOT3* transcripts from WT, *hot3-2*, and *hot3-3*. The gene model and the relative T-DNA insertion sites in *hot3-2* and *hot3-3* are shown below. All three genotypes share the same CPM (Counts Per Million) scale bar.
B. Volcano plots for differentially expressed genes (DEGs) in *hot3-2* and *hot3-3* seedlings. Genes with significant changes (adjusted $P < 0.01$; log2 fold-change (*hot3*/WT) ≥ 1 or ≤-1) are marked in red and blue.
C. Venn diagram to show upregulated DEGs shared by *hot3-2* and *hot3-3.*
D. Gene ontology (GO) term enrichment plot for the 1292 shared upregulated DEGs in *hot3* alleles in (C). Top GO terms of up-regulated DEGs with FDR < 0.01 are shown.

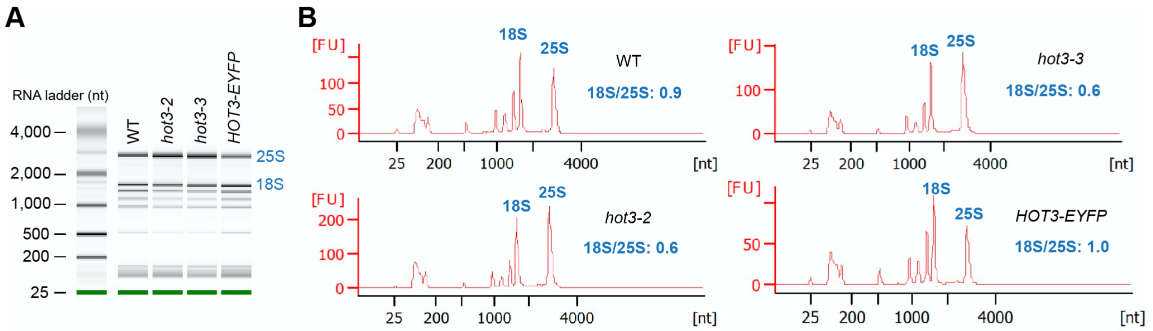


**Fig. S3. The ratio of 18S vs. 25S rRNAs is decreased in *hot3* alleles.**

A. Bioanalyzer analysis for total RNAs from 18-day-old seedlings of WT, *hot3-2*, *hot3-3*, and *HOT3::HOT3-EYFP hot3-2* (*HOT3-EYFP*). The cytoplasmic 25S and 18S rRNAs are indicated in blue text.
B. Quantification of the 18S/25S ratio in each background.

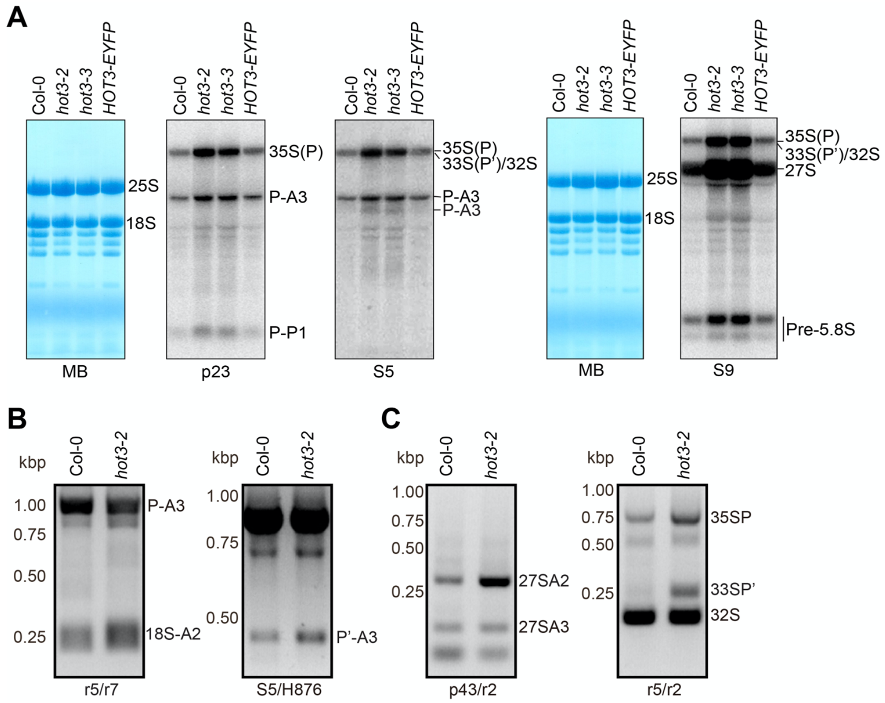


**Fig. S4. Northern blotting and cRT-PCR analyses to characterize rRNAs in WT and *hot3*.**

A. Northern blots with probes p23, S5, and S9 for seedling total RNAs from WT, *hot3-2*, *hot3-3*, and *HOT3::HOT3-EYFP hot3-2* (*HOT3-EYFP*). Blots of p23 and S5 share the same membrane. The methylene blue (MB)-stained membrane is shown on the left.
B-C. Circular RT-PCR analysis to examine pre-rRNAs in WT and *hot3-2.* cDNAs reverse transcribed by the primer 18c2 were amplified to capture pre-18S processing intermediates, including P-A3 and 18S-A2 by r5/r7 primers and P'-A3 by S5/H876 primers (B). cDNAs reverse transcribed by 25c1 were amplified to capture 27S intermediates 27SA2 and 27SA3 by p43/r2 primers, and early pre-rRNA transcripts 35SP, 33SP' and 32S by r5/r2 primers (C). Refer to Fig. 1D for the positions of primers and Fig. S3A for the various pre-rRNA processing intermediates.

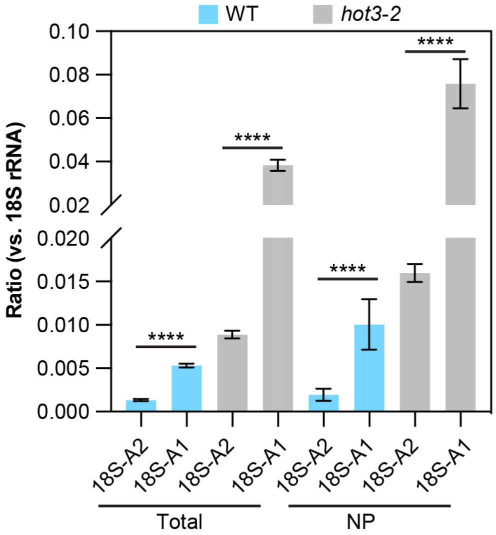


**Fig. S5. Abundance of 18S-A1 and 18S-A2 in total and non-polysome (NP) fractions as determined by 18S-ENDseq.**
The abundance of 18S-A1 or 18S-A2 was normalized against that of 18S rRNA. Mean values of 4 replicates with standard deviation are shown. The unpaired nonparametric t-test was used to evaluate significance of differences between WT and *hot3-2*. ****, *P* <0.0001.

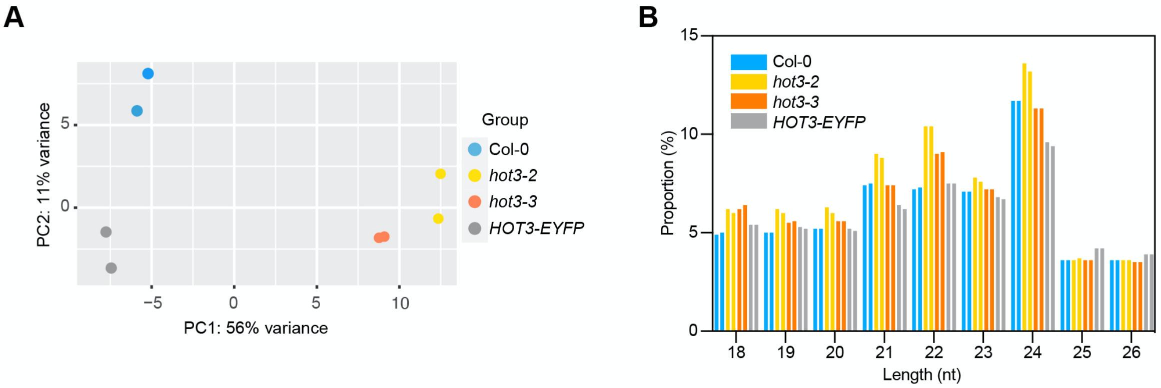


**Fig. S6. Quality control of small RNA sequencing libraries.**

A. Principal components analysis (PCA) analysis of small RNA-seq libraries. Two independent replicates for each genetic background were consistent. Small RNAs assigned to 100-bp non-overlapping windows along chromosomes in each library were used for the PCA analysis.
B. Length distribution of 18-26 nt small RNAs that were mapped to the genome in different backgrounds.

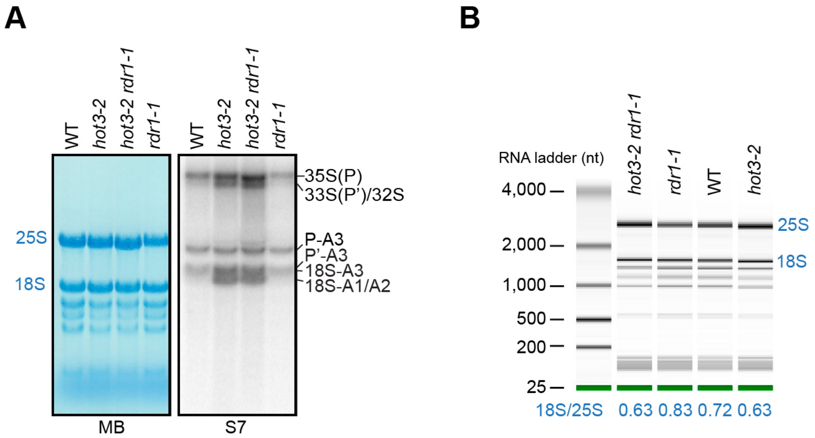


**Fig. S7. Blocking risiRNA biogenesis does not rescue 18S rRNA maturation defects in *hot3-2*.**

A. A northern blot with the probe S7 for seedling total RNAs from WT, *hot3-2*, *hot3-2 rdr1-1*, and *rdr1-1*. The *hot3-2* and *hot3-2 rdr1-1* plants have similar pre-18S processing defects.
B. Bioanalyzer analysis for seedling total RNAs from WT, *hot3-2*, *hot3-2 rdr1-1*, and *rdr1-1*. The ratio of 18S vs. 25S rRNAs is shown below the image. The *hot3-2* and *hot3-2 rdr1-1* plants have reduced levels of 18S rRNAs.

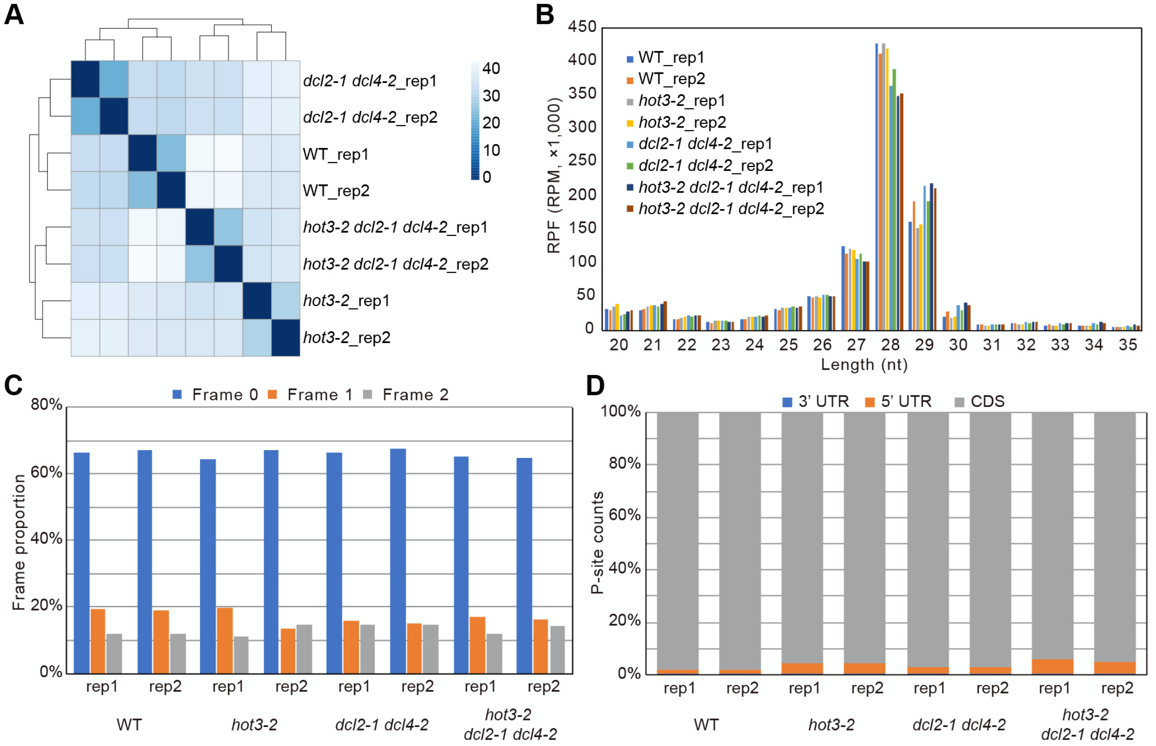


**Fig. S8. Quality of Ribo-seq datasets.**

A. Hierarchical clustering for Ribo-seq datasets. The euclidean distances of samples were calculated by 'regularized log' transformed, normalized P-sites per gene. Rep, independent replicates.
B. Length distribution of normalized RPFs in RPM (reads per million mapped RPFs) in Ribo-seq libraries in this study.
C. Proportions of ribosome protected fragments (RPFs) at each of the three reading frames as revealed by ribosome profiling. RPFs of 26-29 nucleotides from all transcripts were included in the analysis. Frame 0 is the frame of annotated ORFs.
D. Distribution of P-site reads mapped to coding genes.

**Table S1**. List of primers used in this study. All sequences are given from 5′–3′.

| Primer ID | Sequence (5'-3') | Usage |
|---|---|---|
| HOT3g_FP | TCGATAAGCTTGATATCGAATTC AGAGCCGGTTCCAAATTCTTTC | Transgene construction |
| HOT3g_RP | GGCCGCTCTAGAACTAGTGGATCC CTGTATCTTGAAGATGTTCTTCAG | |
| FIB2g_FP | TCGATAAGCTTGATATCGAATTC GGACATACATCGTCGTCTCTAC | |
| FIB2g_RP | GGCCGCTCTAGAACTAGTGGATCCAGCAGCAGTAGCAGCCTTTGGCTTC | |
| S7 | GTCGTTCTGTTTTGGACAGGTATCGA | pre-rRNA Northern blot |
| p23 | GTTCCAACTAATCTACCGAAGTAC | |
| S5 | ATTCCCCGCCACATCCTCTCAAAC | |
| p42 | CCACGGATCCGGCGGGCAAGG | |
| S9 | AGGATGGTGAGGGACGACGATTT | |
| 18c2 | ATGCGTCCCTTCCATAAGTC | cRT-PCR |
| 25c1 | AACGTTAAAAGTAGTGGTATTTCA | |
| r5 | TGCATGGCTTAATCTTTGAGAC | |
| r2 | GCAGACGACTTAAATACGCGAC | |
| r7 | GGAAGGATCATTGTCGATAC | |
| H876 | TTGAATGATCCGGTGAAGTGTTC | |
| p43 | ATGCCAGCCGTTCGTTTGCATG | |
| 18S-P1474 | ACTGATGTATTCAACGAGTTCACA | risRNA Northern blot probe |
| 18S-P1692 | ACGTGGGTGGTTCGCCGCCCG | |
| 18S-P1715 | ACGTCGCGAGAAGTCCACTAA | |
| 18S-P1658 | TTGAATGATCCGGTGAAGTGTTC | |
| 25S-P3333 | GCCACGATCCACTGAGATTCAGC | |
| U6 | AGGGGCCATGCTAATCTTCTCTG | U6 snRNA Northern blot |
| TAS3_5'D7(+) | GGGGTCTTACAAGGTCAAGAA | TAS3_5'D7(+) siRNA Northern blot |
| miR390 | GGCGCTATCCCTCCTGAGCTT | miR390 Northern blot |
| R069 | ACTTACCAGGTCCAGACATAGTAAG | PCR to get 600-bp fragment |
| R070 | CAATGATCCTTCCGCAGGTTC | |
| hot3-2gtLP | ATAAAAGGAGGAGCAGCTTGC | genotyping for hot3-2 (SALK_124251) |
| hot3-2gtRP | TCACCCATGATGACACAAGTG | |
| hot3-3gtLP | GCTTCATTGCCTTCACGATAG | genotyping for hot3-3 (SAIL_1147_G06) |
| hot3-3gtRP | CAGGGGGAAGATGAGGTAGAG | |
| SALK_LB | TGGTTCACGTAGTGGGCCATCG | genotyping LB primer for SALK line |
| SAIL_LB | TAGCATCTGAATTTCATAACCAATCTCGATACAC | genotyping LB primer for SAIL line |
| GABI_LB | ATATTGACCATCATACTCATTGC | genotyping LB primer for GABI line |
| sgs3-14gtLP | AAATTTGGAGTCCAGAATCGG | genotyping for sgs3-14 (SALK_001394) |
| sgs3-14gtRP | CAAAGCATCGGAATCATTCTC | |
| rdr6-15gtLP | GGTTCTCCCTTTTTCGCATAC | genotyping for rdr6-15 (SAIL_617_H07) |
| rdr6-15gtRP | GCTGCAAAATAAGCACAAAGC | |
| rdr1-1gtLP | CCAAGATTTGAGTTGGTTCAGG | genotyping for rdr1-1 (SAIL_672F11) |
| rdr1-1gtRP | GGGAAGTTGTGGATCGTCTCGACGC | |
| dcl2-1gtLP | TGAATCATCTGGAAGAGGTGG | genotyping for dcl2-1 (SALK_064627) |
| dcl2-1gtRP | CTTCACAGGAGTTTTTGGCTG | |
| dcl4-2gtLP | TTTGCCAGTCTTACAAGTGGG | genotyping for dcl4-2 (GABI_160G05) |
| dcl4-2gtRP | GAGGCACCATATAGCAGCTTG | |
| 18S_760 | AATGTATCCAGAGCGTAGGCTTGCTTTGAGCACTCTAATT/3Bio-TEG/ | 18S rRNA depletion |
| 18S_800 | AACACAATAGGATCGAAATCCTATGATGTTATCCCATGCT/3Bio-TEG/ | |
| 18S_80 | CAAACTATAACTGATTTAATGAGCCATTCGCAGTTTCACA/3Bio-TEG/ | |
| 25S_0 | GCTTAAACTCAGCGGGTAATCCCGCCTGACCTGGGGTCGC/3Bio-TEG/ | 25S rRNA depletion |
| 25S_320 | ACCTCGCGGTACTTGTTCGCTATCGGTCTCTCGCCCGTAT/3Bio-TEG/ | |
| 25S_440 | TCGGCGGACCGGATTGCTCCGTTCCGCATCCGACCAGGAC/3Bio-TEG/ | |
| 5.8S_0 | CGATGCGAGAGCCGAGATATCCGTTGCCGAGAGTCGTTTT/3Bio-TEG/ | 5.8S rRNA depletion |
| 5.8S_80 | CGCAACTTGCGTTCAAAGACTCGATGGTTCACGGGATTCT/3Bio-TEG/ | |
| Ch16S_0 | CCAGCGTTCATCCTGAGCCAGGATCGAACTCTCCATGAGA/3Bio-TEG/ | 16S rRNA depletion |
| Ch16S_440 | TACCGCGGCTGCTGGCACAGAGTTAGCCGATGCTTATTCC/3Bio-TEG/ | |
| Ch16S_800 | ACTCCCCAGGCGGGATACTTAACGCGTTAGCTACAGCACT/3Bio-TEG/ | |

| | | |
|---|---|---|
| Ch23S_1240 | CGACATTCTCGCTTCCGCTTCGTCCACCGCCGCTCGCGCG/3Bio-TEG/ | 23S rRNA depletion |
| Ch23S_1920 | CAAGGAATTTCGCTACCTTAGGACCGTTATAGTTACGGCC/3Bio-TEG/ | |
| Ch23S_440 | CCTCACGGTACTACTTCGCTATCGGTCACCCAGGAGTATT/3Bio-TEG/ | |
| P7_Index48 | CAAGCAGAAGACGGCATACGAGATTGCCGAGTGACTGGAGTTCAGACGTGTGCT CTTCCGATCT GAATGATCCGGTGAAGTGTTC | 18S-ENDseq |
| P7_Index47 | CAAGCAGAAGACGGCATACGAGATCTTCGAGTGACTGGAGTTCAGACGTGTGCT CTTCCGATCT GAATGATCCGGTGAAGTGTTC | |
| P7_Index46 | CAAGCAGAAGACGGCATACGAGATTCGGGAGTGACTGGAGTTCAGACGTGTGCT CTTCCGATCT GAATGATCCGGTGAAGTGTTC | |
| P7_Index45 | CAAGCAGAAGACGGCATACGAGATGAATGAGTGACTGGAGTTCAGACGTGTGCT CTTCCGATCT GAATGATCCGGTGAAGTGTTC | |
| P7_Index44 | CAAGCAGAAGACGGCATACGAGATATTATAGTGACTGGAGTTCAGACGTGTGCTC TTCCGATCT GAATGATCCGGTGAAGTGTTC | |
| P7_Index43 | CAAGCAGAAGACGGCATACGAGATGCTGTAGTGACTGGAGTTCAGACGTGTGCT CTTCCGATCT GAATGATCCGGTGAAGTGTTC | |
| P7_Index41 | CAAGCAGAAGACGGCATACGAGATGTCGTCGTGACTGGAGTTCAGACGTGTGCT CTTCCGATCT GAATGATCCGGTGAAGTGTTC | |
| P7_Index39 | CAAGCAGAAGACGGCATACGAGATGTATAGGTGACTGGAGTTCAGACGTGTGCT CTTCCGATCT GAATGATCCGGTGAAGTGTTC | |
| P7_Index38 | CAAGCAGAAGACGGCATACGAGATAGCTAGGTGACTGGAGTTCAGACGTGTGCT CTTCCGATCT GAATGATCCGGTGAAGTGTTC | |
| P7_Index37 | CAAGCAGAAGACGGCATACGAGATATTCCGGTGACTGGAGTTCAGACGTGTGCT CTTCCGATCT GAATGATCCGGTGAAGTGTTC | |
| P7_Index29 | CAAGCAGAAGACGGCATACGAGATTAGTTGGTGACTGGAGTTCAGACGTGTGCTC TTCCGATCT GAATGATCCGGTGAAGTGTTC | |
| P7_Index28 | CAAGCAGAAGACGGCATACGAGATCTTTTGGTGACTGGAGTTCAGACGTGTGCTC TTCCGATCT GAATGATCCGGTGAAGTGTTC | |
| P5 (Uni_r5) | AATGATACGGCGACCACCGAGATCTACACTCTTTCCCTACACGACGCT CTT CCG ATCT TGCATGGCTTAATCTTTGAGAC | |

The abbreviation of 3Bio-TEG represents a biotin modifier with a tetraethyleneglycol (TEG) spacer on the 3′-end of the DNA. It is the same notation used by the DNA supplier (Integrated DNA Technologies, Coralville, IA, USA).

## SI References


1. L. Zhang *et al.*, Mutations in eIF5B Confer Thermosensitive and Pleiotropic Phenotypes via Translation Defects in Arabidopsis thaliana. *Plant Cell* **29**, 1952-1969 (2017).
2. X. B. Wang *et al.*, The 21-nucleotide, but not 22-nucleotide, viral secondary small interfering RNAs direct potent antiviral defense by two cooperative argonautes in Arabidopsis thaliana. *Plant Cell* **23**, 1625-1638 (2011).
3. X. Zhang, R. Henriques, S. S. Lin, Q. W. Niu, N. H. Chua, Agrobacterium-mediated transformation of Arabidopsis thaliana using the floral dip method. *Nat Protoc* **1**, 641-646 (2006).
4. B. Zhang *et al.*, Linking key steps of microRNA biogenesis by TREX-2 and the nuclear pore complex in Arabidopsis. *Nat Plants* **6**, 957-969 (2020).
5. R. Hang *et al.*, Arabidopsis protein arginine methyltransferase 3 is required for ribosome biogenesis by affecting precursor ribosomal RNA processing. *Proc Natl Acad Sci U S A* **111**, 16190-16195 (2014).
6. R. Hang *et al.*, Ribosomal RNA Biogenesis and Its Response to Chilling Stress in Oryza sativa. *Plant Physiol* **177**, 381-397 (2018).
7. G. M. Church, W. Gilbert, Genomic sequencing. *Proc Natl Acad Sci U S A* **81**, 1991-1995 (1984).
8. A. Barkan, Nuclear Mutants of Maize with Defects in Chloroplast Polysome Assembly Have Altered Chloroplast RNA Metabolism. *Plant Cell* **5**, 389-402 (1993).
9. A. Barkan, Proteins encoded by a complex chloroplast transcription unit are each translated from both monocistronic and polycistronic mRNAs. *EMBO J* **7**, 2637-2644 (1988).
10. S. Li, The Isolation of Total and Membrane-Bound Polysomes from Arabidopsis and the Detection of Their Associated AGO1 and sRNAs. *Methods Mol Biol* **1932**, 317-333 (2019).
11. Y. Zhao, S. Li, Isolation of Cytosol, Microsome, Free Polysomes (FPs) and Membrane-bound Polysomes (MBPs) from Arabidopsis Seedlings. *Bio Protoc* **7**, e2436 (2017).
12. R. Hang, X. Deng, C. Liu, B. Mo, X. Cao, Circular RT-PCR Assay Using Arabidopsis Samples. *Bio Protoc* **5**, e1533 (2015).
13. T. Darriere *et al.*, Upon heat stress processing of ribosomal RNA precursors into mature rRNAs is compromised after cleavage at primary P site in Arabidopsis thaliana. *RNA Biol* **19**, 719-734 (2022).
14. I. Unfried, U. Stocker, P. Gruendler, Nucleotide sequence of the 18S rRNA gene from Arabidopsis thaliana Co10. *Nucleic Acids Res* **17**, 7513 (1989).
15. I. Unfried, P. Gruendler, Nucleotide sequence of the 5.8S and 25S rRNA genes and of the internal transcribed spacers from Arabidopsis thaliana. *Nucleic Acids Res* **18**, 4011 (1990).
16. P. Gruendler, I. Unfried, R. Pointner, D. Schweizer, Nucleotide sequence of the 25S-18S ribosomal gene spacer from Arabidopsis thaliana. *Nucleic Acids Res* **17**, 6395-6396 (1989).
17. P. Gruendler, I. Unfried, K. Pascher, D. Schweizer, rDNA intergenic region from Arabidopsis thaliana. Structural analysis, intraspecific variation and functional implications. *J Mol Biol* **221**, 1209-1222 (1991).
18. P. Y. Hsu *et al.*, Super-resolution ribosome profiling reveals unannotated translation events in Arabidopsis. *Proc Natl Acad Sci U S A* **113**, E7126-E7135 (2016).
19. I. V. Kim *et al.*, Efficient depletion of ribosomal RNA for RNA sequencing in planarians. *BMC Genomics* **20**, 909 (2019).
20. M. Martin, Cutadapt removes adapter sequences from high-throughput sequencing reads. *EMBnet.journal* **17**, 3 (2011).
21. A. Dobin *et al.*, STAR: ultrafast universal RNA-seq aligner. *Bioinformatics* **29**, 15-21 (2013).
22. Y. Liao, G. K. Smyth, W. Shi, featureCounts: an efficient general purpose program for assigning sequence reads to genomic features. *Bioinformatics* **30**, 923-930 (2014).
23. L. Calviello *et al.*, Detecting actively translated open reading frames in ribosome profiling data. *Nat Methods* **13**, 165-170 (2016).
